# Dietary and Nutritional Factors in Systemic Lupus Erythematosus Pathophysiology: A Scoping Review of the Evidence from *In Vitro*, *In Vivo,* and Human Studies

**DOI:** 10.1016/j.advnut.2026.100644

**Published:** 2026-05-06

**Authors:** Justin N Jaya, Michael Pudjihartono, Nicholas Pudjihartono, Ivan Damara, Fahrul Nurkholis, Felix Zulhendri

**Affiliations:** 1 Faculty of Medicine, Universitas Indonesia, Jakarta, Indonesia; 2 Liggins Institute, The University of Auckland, Auckland, New Zealand; 3 Richmond University Medical Center, Staten Island, NY, United States; 4 Master of Basic Medical Science, Faculty of Medicine, Universitas Airlangga, Surabaya, Indonesia; 5 State Islamic University of Sunan Kalijaga (Universitas Islam Negeri Sunan Kalijaga), Yogyakarta, Indonesia; 6 Medical Research Center of Indonesia, Surabaya, Indonesia; 7 Kebun Efi, Sumatera Utara, Indonesia; 8 Department of Pharmacology and Clinical Pharmacy, Faculty of Pharmacy, Universitas Padjadjaran, Sumedang, Indonesia

**Keywords:** systematic lupus erythematosus, autoimmune disease, dietary factors, nutrition, scoping review

## Abstract

Systemic lupus erythematosus (SLE) is a complex autoimmune disease whose pathogenesis involves an interplay between genetic predisposition and environmental factors, including diet. This scoping review maps and synthesizes current evidence from human and animal studies on the relationships between dietary factors and lupus pathophysiology. Following PRISMA guidelines, we identified 139 relevant studies from Scopus, PubMed, and EBSCO published between 2012 and 2023. Our analysis reveals that specific dietary components significantly influence lupus risk and disease activity. Diets high in sugar, carbohydrates, and sodium were associated with increased inflammation and exacerbated disease severity. Conversely, several factors demonstrated protective effects. Higher intake of omega (ω)-3 PUFAs was consistently linked to reduced inflammatory markers and improved patient-reported outcomes, whereas a higher ω-6 to ω-3 ratio correlated with worsened disease activity. Moderate alcohol consumption, particularly wine, was associated with a reduced risk of SLE incidence. Adequate vitamin D concentrations were connected to attenuated disease progression and immunomodulation. Furthermore, natural products like olive oil phenolic compounds and curcumin showed promise in reducing oxidative stress and inflammatory pathways in murine models. The evidence underscores that dietary modification presents a viable strategy for modulating immune function and inflammation in SLE. Integrating nutritional guidance with conventional therapies could improve disease management. Future large-scale randomized controlled trials are essential to establish precise dietary recommendations and elucidate the mechanisms underlying diet–lupus interactions.

**Statement of Significance**

This scoping review offers a comprehensive synthesis of the evidence linking dietary and nutritional factors in systemic lupus erythematosus (SLE) pathophysiology.

## Introduction

Systemic lupus erythematosus (SLE) is an autoimmune disease characterized by extensive dysfunction of both the adaptive and innate immune systems [1]. SLE can manifest clinically in various organs, including the skin (cutaneous lupus erythematosus), kidneys (lupus nephritis), joints, lungs, cardiovascular system, and central nervous system [2]. In SLE, autoreactive B cells, supported by T cells,produce a variety of autoantibodies, including antinuclear antibodies (ANA), anti-Smith antibodies, and antibodies against double-stranded DNA (dsDNA), which can form immune complexes that are deposited in various organ tissues [3,4]. However, antibody deposition is not the only complication involving the immune system in SLE. For example, dysregulation in the complement system has also been documented, leading to an amplified inflammatory response that exacerbates tissue damage [5,6]. As a result, the management of lupus often involves multitarget therapies using immunosuppressive agents and corticosteroids [7].

The exact causes of SLE are not fully understood, but it is believed to involve both genetic and environmental factors [8,9]. The role of environmental factors is highlighted by the significantly higher SLE incidence in African Americans compared with West Africans, despite their shared genetic background [10]. The mechanisms underlying this disparity remain unclear, but dietary factors may play a role. Indeed, dietary and lifestyle components can modulate immune responses and the inflammatory processes that underlie lupus pathogenesis.

For example, micronutrients such as vitamin D and zinc have emerged as key regulators of immune function, oxidative stress, and inflammation [[11], [12], [13]]. Additionally, dietary plant secondary metabolites such as polyphenols have shown promise in modulating immune responses, gut microbiome, and mitigating inflammatory processes implicated in lupus pathogenesis [14].

We hypothesized that specific dietary and nutritional factors are linked to lupus risk and disease activity, with some being protective and others harmful. Given growing evidence connecting diet, micronutrients, and immune modulation, this review aims to synthesize findings from observational studies, *in vitro*, animal models, and human clinical data on diet–SLE interactions.

Unlike prior reviews that focus on single nutrients, this study offers several contributions. First, it integrates evidence from human studies and animal models to provide mechanistic insight alongside epidemiological data. Second, it organizes the literature into key categories such as macronutrients, essential fatty acids, vitamin D, sodium, alcohol, and natural products, and therefore allows comparison of protective and harmful factors. Third, it includes emerging evidence on bioactive compounds such as olive oil phenolics and curcumin, which have received limited attention. Overall, this approach provides a more holistic view of how dietary patterns, rather than single nutrients, influence lupus pathogenesis.

## Methods

**FIGURE 1:**
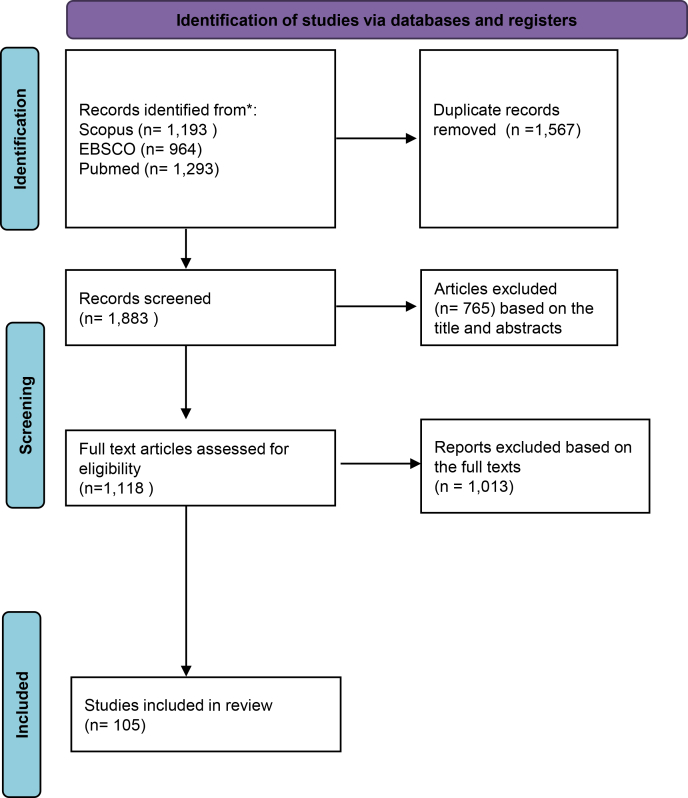
The flow chart of the study identification and selection process.

The systematic review was conducted according to the PRISMA [15]. The 4-phase flow PRISMA methodology was followed, as seen in Figure 1 [16]. The search for peer-reviewed manuscripts published between 1 January, 2012 and July 2023 was conducted by 4 authors independently (FZ, NP, MP, and JJ). The Scopus, PubMed, and EBSCO databases were queried between 1 January, 2012 and 9 July, 2023. The search terms that were used were: lupus and [(food) or (diet) or (nutrition∗) or (nutrient∗)].

Studies were included if they met the following criteria: *1*) primary research articles (observational studies, clinical trials, *in vivo* animal studies, and *in vitro* mechanistic studies); *2*) investigation of an association between dietary/nutritional factors and SLE outcomes (incidence, disease activity, biomarkers, organ involvement); *3*) published in peer-reviewed journals; *4*) English language; *5*) published between January 2012 and July 2023. Dietary factors were defined as any nutritional component consumed orally, including macronutrients (carbohydrates, proteins, and fats), micronutrients (vitamins and minerals), bioactive compounds (polyphenols and flavonoids), whole foods, dietary patterns, and nutritional supplements. Exclusion criteria included narrative reviews, systematic reviews, meta-analyses, commentaries, conference abstracts, editorials, and studies not specifically addressing SLE.

The included studies were documented in Mendeley, and duplicate records were eliminated. Studies were evaluated by a comprehensive examination of titles, keywords, abstracts, and full texts. Articles that did not align with the guiding statement and the predetermined criteria were subsequently excluded. In the event of disagreement regarding the eligibility of a specific article, resolution was achieved through discussion with all authors involved in the initial search; the study was included only if all authors agreed. Summaries of included studies were collected and organized in Microsoft Excel. Finally, the authors classified studies into distinct thematic categories: macronutrients, ultraprocessed foods and general dietary factors, alcohol consumption, iodine intake, vitamin D, essential fatty acids, and natural products. In this review, “general dietary factors” encompass overall dietary patterns, macronutrient composition, ultraprocessed food consumption, and lifestyle factors that influence nutritional status (e.g., meal patterns, dietary quality indices, and eating behaviors).

## Discussion

### Macronutrients and general dietary factors

Macronutrients and general dietary factors have been extensively studied in relation to SLE, offering insights into the role of diet in disease activity and risk. Correa-Rodríguez et al. [17] explored the association between dietary sugar intake and cardiovascular disease risk markers in patients with SLE. The study included 193 patients and examined clinical and metabolic factors like disease activity, obesity, diabetes, hypertension, and lipid concentrations. It found that higher intake of free sugars was linked to greater disease activity and complications, including dyslipidemia [17]. This suggests that elevated sugar consumption may exacerbate disease progression and cardiovascular disease risk in patients with SLE.

Additionally, the composition of macronutrients in the diet has been examined for its potential influence on SLE risk. Castro-Webb et al. [18] analyzed data from the Black Women’s Health Study and found that higher intakes of MUFAs, saturated fats, and *trans*-fats were linked to a decreased risk of developing SLE. Interestingly, a diet high in carbohydrates, particularly from sugar-sweetened beverages and fruits, and low in fats, was associated with an increased risk of SLE. These findings suggest that a balanced intake of fats, particularly MUFAs and saturated fats, may offer some protective effects against SLE onset [18].

Julià et al. [19] added further evidence to the role of sugar intake in SLE by showing that patients with SLE consumed higher levels of sweets compared with healthy controls, highlighting a possible dietary pattern that could influence disease activity. Similarly, Petrić et al. [20] investigated the dietary habits of 76 patients with SLE in clinical remission and discovered associations between low-quality proteins, calorie-rich foods, and reduced concentrations of complement proteins C3 and C4 [20]. These proteins are crucial in immune regulation, and their decreased concentrations could signal potential disease activation, suggesting that diets high in fast food and fried foods may contribute to disease flares [20].

On a broader scale, lifestyle factors also play a significant role in SLE risk. Choi et al. [21] conducted an extensive analysis of 185,962 females over 4,649,477 person-years from the Nurses’ Health Study (NHS) and NHSII cohorts, examining the relationship between a Healthy Lifestyle Index Score and SLE incidence. They found that adopting healthy lifestyle practices, such as maintaining a balanced diet, regular physical activity, and a healthy body weight, could halve the risk of developing SLE, despite the known genetic predisposition to the disease [21]. These findings reinforce the importance of modifiable lifestyle factors in mitigating disease risk.

Nutritional status itself has also been linked to disease severity in patients with SLE. Correa-Rodríguez et al. [22] highlighted that poor immune-nutritional status, as measured by the Prognostic Nutritional Index and Nutritional Risk Index, correlated with heightened disease activity and organ damage in patients with SLE. Behiry et al. [23] reported that over three-quarters of the patients with SLE in their study were either overweight or obese, with their dietary patterns characterized by reduced intake of fruits, vegetables, and dairy products, and elevated consumption of fats and oils. This unhealthy dietary pattern was associated with increased BMI, body weight, and disease duration, indicating a direct link between poor dietary choices and disease progression [23].

Contradictory evidence regarding the role of diet in SLE risk has also emerged. Tedeschi et al. [24] examined the relationship between dietary quality and SLE risk in the NHS and NHSII cohorts and found no significant association between prudent or Western dietary patterns and SLE incidence. Similarly, Barbhaiya et al. [25] evaluated the impact of 4 dietary quality scores; namely, the Alternative Healthy Eating Index-2010, Alternative Mediterranean Diet Score, Dietary Approach to Stop Hypertension, and Empirical Dietary Inflammatory Pattern, and found that long-term adherence to these dietary patterns did not significantly influence SLE risk.

### Animal studies suggest modes of action of how general dietary factors may affect the pathophysiology of lupus

In Toll-like receptor 7 (TLR7)-dependent mouse model, a Western-style diet appears to be linked to the incidence of lupus through changes in the microbiota composition [26]. In TLR8-deficient mice, a high-fat diet worsens lupus through TLR7 signaling. This leads to stronger immune responses, more anti-DNA antibodies, increased IgG/IgM deposits in the kidney, and greater kidney damage [27]. Table 1 [17,[22], [23], [24],[26], [27], [28], [29], [30], [31], [32], [33], [34], [35], [36], [37], [38], [39], [40], [41], [42], [43], [44], [45], [46], [47], [48], [49], [50], [51], [52], [53], [54], [55], [56], [57], [58], [59], [60], [61], [62], [63], [64], [65], [66], [67], [68], [69], [70], [71], [72], [73], [74] summarizes the effect of macronutrients, ultraprocessed foods, and general dietary factors on lupus incidence and severity.

**TABLE 1:** The effect of macronutrients, ultraprocessed foods, and general dietary factors on lupus incidence and severity

Study design	Organism	Interventions/nutrient	Outcome measured	Study result	Reference
Case control	Human	—	• Risks for SLE and MCTD	• Smoking and walking for a longer time showed an increased age-adjusted risk for MCTD as well as SLE. • Frequent intake of bread increased the risk of MCTD, and high intake of green tea decreased the risk of MCTD. • Westernization of dietary habits (i.e., frequent intake of bread and low intake of green tea) may increase the risk of MCTD, whereas walking may increase the risk of SLE among Japanese females.	[28]
Case control	Human	—	• Prevalence of SLE • Gender difference in the prevalence of SLE • Age-specific prevalence of SLE • Environmental Factors Associated With SLE	• The estimated prevalence of SLE in rural areas of Anhui Province was 37.56 per 100,000 persons. • There was a significant gender difference in the prevalence of SLE, with a higher prevalence among females. • Several factors were associated with an increased risk of SLE, including preterm/post-term births, consumption of sweet food, animal oil, preference for light taste, sunlight exposure, insufficient sleep, lack of physical activities, drinking water from a pond or well, experiencing a negative life event, receiving hepatitis B vaccination, and age at birth of first child. • Fruit consumption, residing in a flat area, and age at menarche were associated with a reduced risk of SLE.	[29]
Randomized, double blind, placebo-controlled, crossover trial	Human	Creatine	• Muscle function [assessed by a battery of tests including 1 maximum repetition (1-rm) tests, the timed up and go test, the timed stands test, and the handgrip test] • Body composition • Biochemical markers of bone remodeling • Aerobic conditioning • Quality of life • Physical capacity • Muscle phosphoryl creatine content	• Intramuscular phosphoryl creatine content was not significantly different between creatine and placebo before or after the intervention. • No significant changes between placebo and creatine for any muscle function and aerobic conditioning parameters, lean mass, fat mass, bone mass, and quality of life scores. • No side effects were noticed with creatine supplementation.	[30]
Case report	Human	—	• Severe dental erosion and carries	• The consumption of carbonated soft drinks and the presence of bulimia are widely recognized factors contributing to teeth degradation in wealthy nations. The adoption of a Western diet in the Pacific region has resulted in a rise in the prevalence of tooth caries and erosions. The individual’s pronounced dental erosion can be attributed to the concurrent presence of xerostomia and the habitual consumption of limes for alleviation.	[31]
Case control	Human	—	• SLE disease activity (SLEDAI) • Ceruplasmin, albumin (Alb), and trace elements including zinc (Zn), copper (Cu), and selenium (Se). • Antidouble-stranded DNA (anti-dsDNA), complement serum values including C3, C4, creatinine, full blood count, urinary sedimentation (RBC or WBC cast), and 24 h urine protein excretion	• Serum concentrations of Alb, Zn, Cu, and Se were lower in patients with SLE than in healthy controls. • Serum Alb and Cu concentrations were negatively correlated with lupus disease activity. • The Zn to Cu ratio (Zn/Cu R) was also lower in patients with SLE than in healthy controls.	[32]
Case control	Human	Fasting	• SLE Disease Activity Index (SLEDAI) • Lipid Profile • Quality of Life with Short Form 36 (SF-36) Health Survey	• Ramadan fasting had no effect on disease activity or quality of life in patients with SLE during the quiescent phase of disease. • Anti-ds DNA and C3 increased in cases after 24.1 ± 5.4 d of fasting but returned to baseline after 3 mo. • Total cholesterol decreased in cases after Ramadan fasting, but not in controls.	[33]
Case report	Human	Gluten-free diet	• Presence and intensity of auditory and visual hallucinations • Depressed mood • Social withdrawal • Academic performance • Presence of antinuclear antibodies (ANA) • Gluten sensitivity • Psychotic symptoms • Dosage of risperidone • Anxiety • Hearing loss • Autoimmune inner ear disease	• Improvement in psychotic symptoms after a gluten-free diet in a boy with complex autoimmune illness	[34]
Retrospective analysis	Human	—	• Fasting blood glucose (FBG) • Levels of diseases: # of tests, mean, median, –log10 p value	• 57/64 diseases including type 2 diabetes, pancreatitis, diabetic nephropathy, and pancreatic cancer had significantly increased FBG concentrations compared with healthy controls. • 6/64 diseases including preeclampsia, Wilms’ tumor, and lupus erythematous had significantly decreased FBG concentrations compared with healthy controls. • Increased FBG concentrations might be a consequence but not the cause of either prediabetes or type 2 diabetes.	[35]
Observational and *In vivo* trial	Human and rat	Animal study: Choline-rich diet, LPS, and cultured in interferon (IFN)-γ or IL-4 and IL-13	• Multivariate statistical analysis of 1H NMR spectrum of human urine • Integral area of corresponding metabolites comparing healthy, SLE, and patients with RA: TMAO (trimethylamine-N-oxide), Dimethylamine, Citrate, Succinate, p-Cresol sulfate • Patient characteristics: age, gender, hypertension, diabetes mellitus, symptom duration, RF positive, ACPA positive, ESR, CRP, Prednisolone, NSAIDs, Methotrexate, Hydroxychloroquine, Anti-TNF-α • Cluster correlation analysis: ESR, DAS28, CRP, IL-6. CCL2, IL-8, WBC, p-CD, citrate, succinate, DMA, TMAO, Hb, Albumin • Comparison of urinary metabolite concentrations: succinate, TMAO, citrate, p-Cresol sulfate, dimethylamine with ESR, CRP, DAS28, Hb • Radiographic progression: TMAO, dimethylamine, p-Cresol sulfate, citrate, succinate • Multivariate logistic regression analysis odds ratio: HCQ, p-CS, disease duration, IL-6 • Arthritis score • Relative expression: iNOS, Arg1, IL-6	• Patients with RA and SLE showed a distinct urinary metabolomics pattern, with markers of altered gut microbiota (TMAO) and oxidative stress (dimethylamine) upregulated, and markers of mitochondrial dysfunction (citrate and succinate) and metabolic waste products (p-CS) downregulated. • TMAO and dimethylamine were negatively associated with serum inflammatory markers in patients with RA. • A choline-rich diet reduces experimentally induced arthritis, suggesting that the interaction between diet and the intestinal microbiota contributes to the RA phenotype.	[36]
Case control	Human	—	• Subject characteristics: survival status, sex, age at onset of disease, age, days because diagnosis, SLEDAI score, thrombocytopenia status, ANA test, anti-dsDNA test, nutritional status • Univariate and multivariate analysis of risk factors and predictors of mortality: hypertension, seizures, proteinuria, SLEDAI score, Infection	• 84 children with SLE were included in the study, of which 72 were female. Most subjects were well-nourished (*n* = 62), whereas somewhere moderately malnourished (*n* = 14), and few were severely malnourished (*n* = 8). • Hypertension and infection were significantly associated with mortality on bivariate analysis. • On logistic regression analysis, infection was the only significant predictor of mortality.	[37]
Observational	Human	—	• Descriptive characteristics of patients: sex, gender, height, weight, BMI, antimalarial, immunosuppressor, corticoid use, total cholesterol, albumin, lymphocyte count, anti-dsDNA, complement C3, C4, WBC count, platelet count, hsCRP, SLEDAI score, SDI score, PNI (prognostic nutritional index), CONUT (controlling nutritional status), NRI (nutritional risk index) • Linear regression and logistic regression analysis between clinical disease activity variables and immunonutritional indexes and SLEDAI and SDI scores: hsCRP, anti-dsDNA, C3, C4, WBC count, platelet count, PNI, CONUT, NRI	• PNI and NRI were significantly lower in active patients with SLE than in inactive patients with SLE. • PNI was inversely correlated with the SLEDAI score, and NRI positively correlated with SLEDAI and SDI scores. • PNI and NRI were independent predictors of active SLE.	[22]
Multicenter	Human	—	• Prevalence of CD in diseases: SLE, primary Sjögren’s syndrome (pSS), systemic sclerosis (SSc) • Epidemiological features comparing CD and healthy control population: total subjects, age, gender, previously diagnosed CD and prevalence, subclinical CD, overall CD prevalence, age at autoimmune diagnosis (AD) • Epidemiological features of CD related to SSc (diffuse or limited subset): total subjects, age, previously diagnosis CD and prevalence • Age of symptom onset	• CD prevalence is significantly increased in primary Sjögren’s syndrome (pSS) and diffuse cutaneous form of systemic sclerosis (SSc) when compared with the general population. Although SLE associations with CD remain uncertain as they trend toward significance (*P* = 0.058). • Subclinical CD was found in 2 patients with SLE and 1 patient with pSS. • CD diagnosis usually preceded that of AD, and autoimmune thyroiditis was associated with pSS and CD.	[38]
Cross-sectional	Human	—	• Demographics and clinical characteristics: age, education level, occupation status, residence (urban/rural), disease duration, age at onset of diagnosis • Anthropometric, medical, and laboratory characteristics: Weight, height, BMI, hemoglobin, anemic, TLC (total lymphocyte count), albumin, steroid duration, SLEDAI score • Daily caloric and macronutrient intake: energy (kcal), proteins, total fats, dietary fibers • Daily iron and calcium intake • Associations between demographics, medical, and laboratory variables with BMI: education, occupation, residence, age, age at disease onset, disease duration, hemoglobin, TLC, albumin, steroid duration, SLEDAI score • SLEDAI score association with anthropometric and laboratory investigation: age, disease duration, age at onset of diagnosis, weight, height, BMI, Hb, TLC, albumin, steroid duration	• More than three-quarters of patients with SLE were overweight and obese. • Disease activity (SLEDAI) correlated with increased BMI, body weight, and disease duration. • Inadequate nutrient intake and excessive consumption of lipids and low intake of fibers were revealed.	[23]
Survey based	Human	—	• Demographics: sex, age • Responses by date: cumulative number of responses, weeks • Survey responses regarding patient opinion on diet in and out of clinic • Age group response comparison • Survey responses regarding patient experiences with diet including reasons, types, and outcomes • cText analysis of dietary modifications and reasonings behind changes, alongside observed benefits • Text analysis of responses thought to should be investigated	• Patients with SLE reported a lack of clinical counseling regarding diet but would be willing to change their diet if they knew it would help their symptoms. • Patients expressed an interest in using diet to treat fatigue and manage disease flares. • An anonymous social media platform was used to successfully gather patient information regarding diet and SLE over a short timeframe.	[39]
Observational	Human	—	• Demographic data: age, sex, follow-up duration • SLE activity-related measures: SLEDAI-2K, WBC count, platelet count, C3, C4, Anti-dsDNA, urinary P/Cr ratio • Clinical features: skin rash, photosensitivity, oral ulcer, arthritis, serositis, neurologic disorder, hematologic disorder, immunologic disorder • Laboratory data: lymphocyte count, ESR, CRP, Cr, GFR, total cholesterol, serum albumin, AST, ALT • Renal biopsy data lupus nephritis class: class I,II, pure class III, IV, V, mixed class V, class V + II, V + III, V + IV, VI • Activity and Chronicity index • Nutritional indices: CONUT (controlling nutritional status) score, PNI, NRI, NLR (neutrophil to lymphocyte ratio), BMI • Correlation analysis between variables and nutritional indices at lupus nephritis diagnosis: SLE activity-related measures, laboratory data, renal biopsy data with nutritional indices • Correlation analysis between nutritional indices and lupus nephritis subclasses at diagnosis • Receiver operator characteristic curve of nutritional indices at lupus nephritis diagnosis in predicting end-stage renal failure: Sensitivity against 100-Specificity • Kaplan-Meier curve analysis of renal survival rate based on PNI • Cox-proportional hazard analysis of variables associated with end-stage renal failure during follow-up (univariate and multivariate analysis): SLE activity-related measures, laboratory data, nutritional indices	• The CONUT score and PNI had the highest correlation between the SLE disease activity index-2000. • PNI was independently associated with ESRF along with creatinine and chronicity index. • The renal survival rate was significantly lower in patients with PNI ≤35.41 than in those with PNI >35.41.	[40]
Case report	Human	Gluten-free dietary regimen	• Congenital and acquired thrombophilia • Lupus anticoagulant • Antiphospholipid syndrome (APS) diagnosis • Coeliac disease screening • APGAR score • Screening for adverse pregnancy outcomes • Patient immunological status	• Successful pregnancy and lupus anticoagulant remission after gluten-free diet in a female affected by coeliac disease and antiphospholipid syndrome. • Coeliac disease is associated with other comorbidities, including autoimmune conditions and extraintestinal manifestations. • Strict adherence to a gluten-free diet is the only treatment for coeliac disease.	[41]
Cross-sectional	Human	—	• Demographics: BMI, gender, socioeconomic, CVD risk, physical activity, waist/height ratio, body fat % • Dietary intake: total fat, saturated fat, *trans*-fat, cholesterol, carbohydrate • Biomarkers of lipid metabolism: lipid profile, apolipoproteins AI/B, triglycerides, HDL, LDL, C-reactive proteins (CRP), fasting blood glucose	• Overweight and dyslipidemia were observed in a high proportion of both parents and children with chronic rheumatic diseases (80% similarity), indicating an environment conducive to chronic noncommunicable disease development. • There was a moderate association between total fat intake and a weak association between saturated fat intake and cholesterol intake between parents and their children. • There was a weak association between parents and children for triglycerides, but no association between parents and children concerning physical activity.	[42]
Retrospective cohort	Human	—	12 Organ specific morbidities comorbidities: • Neoplasms (malignant and benign) • Blood disorders • Endocrine, nutritional and metabolic disorders • Mental and behavioral disorders • Nervous system disorders • Circulatory system disorders • Other chronic pulmonary diseases • Noninfective enteritis and colitis • Musculoskeletal diseases • Genitourinary diseases • Rate ratios of comorbid diseases and CVD • Gender • Education level	• 91.2% of patients with SLE had ≥1 comorbidity, compared with 66.7% of comparators. • Musculoskeletal, cardiovascular and genitourinary conditions were the most common comorbidities. • “Men with SLE had a significantly higher risk for diseases of the genitourinary system and endocrine, nutritional and metabolic diseases compared with women with SLE.”	[43]
Cross-sectional observational	Human	—	• Demographics: gender, age, disease duration, weight, height, BMI • Concentrations of dietary C3 And C4 • 24-h proteinuria	• Majority of patients had a normal BMI and worked out regularly. • Decreased values of C3 were found in patients who often consumed meat, and decreased values of C4 in patients who often consumed fast food. • Dietary habits can influence the disease course of SLE.	[44]
Retrospective cross-sectional	Human	—	• Demographics, clinical, and laboratory features: gender, age, disease progression, BMI, Z-score height for age, Z-score BMI, nutritional status, SLEDAI, SLICC, CRP, ESR, corticosteroid dose, biologics, and nonbiologics • Lipid profile: triglycerides (TG), total cholesterol (TC), LDL cholesterol, HDL cholesterol, VLDL-C, non-HDL cholesterol (NHDL-C), dyslipidemia	• Dyslipidemia is common in children and adolescents with ARDs, especially JIA, jSLE, and JDM. • The most common alteration in the lipid profile of these patients was decreased HDL cholestrol. • Systemic JIA and cumulative corticosteroid dose in jSLE were associated with an increase in LDL cholesterol and a decrease in HDL cholesterol, respectively.	[45]
Cross-sectional	Human	—	• Demographics: gender, age, urban area, census region, race, smoking, BMI, health conditions, metabolic syndrome • Biochemistry profile: Serum alanine aminotransferase, aspartate aminotransferases, albumin, bicarbonate, alkaline phosphatase, lactate dehydrogenase, total protein, globulin, creatinine, cholesterol, glucose, total bilirubin, calcium, CRP, FSH, LH, transferrin saturation, urinary albumin, cadmium, creatinine, iodine • Nutrition biomarkers: plasma fibrinogen, serum β-carotene, beta cryptoxanthin, cholesterol, lutein/zeaxanthin, selenium, vitamin A, vitamin B-12, vitamin C, vitamin E • Antibody tests: serum rubella antibody, tetanus antibody, toxoplasmosis antibody, latex antibody, hepatitis A, hepatitis B	• Prevalence of lupus in the United States was 241 per 100,000. • Risk factors for lupus included smoking, elevated serum concentrations of chloride, globulin, lactate dehydrogenase, uric acid, cholesterol, and lutein or zeaxanthin. • Protective factors against lupus included non-White race, obesity, elevated serum concentrations of bicarbonate, creatinine, total calcium, and vitamin B-12, as well as elevated urinary albumin and iodine.	[46]
Cross-sectional	Human	—	• Demographics: gender, age, BMI • Clinical data: # of complications, SLEDAI-2K, SDI Damage Index, hsCRP (C-reactive protein), Hcy (homocysteine), anti-dsDNA, complement C3, complement C4 • Medication used: antimalarials, immunosuppressors, corticoids • Cardiovascular disease risk factors: hypertension, diabetes, obesity, dyslipidemia, TC (total cholesterol), TG (triglycerides), HDL cholesterol, LDL cholesterol, SBP (systolic blood pressure), DBP (diastolic blood pressure), ABI (ankle brachial index) • Nutrients: energy, total carbohydrates, proteins, fats, starch, total sugars, intrinsic sugars, free sugars	• A cross-sectional study of 193 patients with SLE revealed a significant association between free sugars intake and disease activity, number of complications, and SLEDAI. • Patients with active SLE had significantly lower dietary sugar intake than those with inactive SLE. • Free sugars intake was associated with increased CVD risk markers.	[17]
Prospective cohort	Human	—	• Demographics: age, race, BMI, smoking, physical activity, household income, oral contraceptive, menopausal status, energy intake, symptom diagnosis, organ system involvement • Anti-dsDNA/SLE risk	• A higher prudent dietary pattern score was not associated with SLE risk. • A higher Western dietary pattern score was not associated with SLE risk. • Incident anti-dsDNA positive SLE and anti-dsDNA negative SLE were not associated with either dietary pattern.	[24]
[47]	Human	—	• Demographics: gender, age, weight, height, BMI, nutritional status • Mexican MEX-SLEDAI • Laboratory test: C3, ANA test, Anti-dsDNA, Hb, WBCs, PLTs • Organ manifestation and disease activity: skin, hematology, renal, neurological, hepar, musculoskeletal	• Malnutrition is still high in jSLE, affecting 45.16% of all subjects. • Malar rash and bicytopenia were significantly higher in active jSLE than inactive. • Renal manifestation was correlated with active jSLE and had the highest risk in active jSLE.	[48]
Cross-sectional association	Human	—	• Patient characteristics: gender, age, disease duration SLEDAI, C3, C4, anti-dsDNA, medications, vitamin B-6, vitamin B-9, vitamin B-12 • CpG17, CpG22, ad CD40L methylation status in T cells • Micronutrient intake: methionine, folate, choline, cysteine, vitamin B-6, and vitamin B-12 • Dietary product intake: pizza, dairy products, chips/French fries, cooked potatoes, white bread, beer, fruit/herbal tea, meat, ice cream	• Dietary methyl donors and products were associated with CD40L methylation status in patients with SLE. • Hypomethylation of CpG17 and CpG22, and not CD40L, was associated with increased disease activity in patients with SLE (SLEDAI). • Dietary products with the highest impact on methylation included meat, ice cream, white bread, and cooked potatoes.	[49]
Observational multicenter	Human	—	• Type/frequency of rheumatic disease: rheumatoid arthritis, SLE, Behçet’s disease, systemic sclerosis, ankylosing spondylitis, psoriatic arthritis, dermatomyositis, relapsing polychondritis, familial Mediterranean fever, adult onset Still’s disease, ulcerative colitis • Nutritional deficiency indicators: wasting, skin rash, hair/nail changes, nail spooning, night blindness, glossitis, peripheral edema, tetany, active LOM (limitation of movement), passive LOM, GI, thyromegaly, weight loss, anorexia • Demographics: gender, age, disease duration, medication usage	• Nutritional deficiency is common among patient with RD, especially elderly. • Type and duration of RD did not significantly affect symptoms or signs of nutritional deficiency. • Age, azathioprine, methotrexate, and hydroxychloroquine were associated with nutritional deficiency manifestations.	[50]
Observational	Human	—	• Patient characteristics: gender, SLICC, SLEDAI, BMI, body fat %, waist circumference Daily intake of micronutrients • Association of metabolic risk and insufficient micronutrient intake (choline, sulfur, and vitamin B-9) • Prevalence of metabolic syndrome (LDL, HDL, cholesterol, and triglycerides)	• A total of 77 patients (68.8%) patients presented a score of 0 in the SLICC, similar to scores for disease activity, with 73 patients (62.5 %) presenting a score of 0 in the SLEDAI. • MR was present in 58 patients (51.8%) who showed an association between MR and low dietary intake of vitamin B-9, choline, and sulfur • 37.5% of patients were classified with degree II obesity by BMI and 76.8% by abdominal obesity.	[51]
Case series	Human	—	• Patient characteristics: gender, family history, age of suspected cSLE, identified genetic variations • SLIICC • Laboratory data: WBC, lymphocytes, hemoglobin, platelets, anti-dsDNA Ab, C3, C4, ANA, proteinuria, hematuria • Symptoms and comorbidities: growth and development, CNS, cardiovascular, gastrointestinal, renal, metabolic, mucocutaneous, musculoskeletal, serology, invasive infections • Treatment: prednisolone, cyclophosphamide, azathioprine, cyclosporin, MMF/MPA, hydroxychloroquine, other treatments • Anthropomorphic observations • Brain changes (CT scan) • Genomic sequencing • Proteinuria (urine protein/creatinine ratio)	• Genetic etiologies and lupus mimickers were found among a substantial proportion of patients suspected with early-onset SLE. • Detail clinical evaluation and genetic testing are important for tailored care and personalized treatment. • Hypoproteinemic diets were given to a patient with cSLE as an alternative for immunosuppressants, sometimes accompanied by vitamin supplementation such as citrulline and/or carnitine.	[52]
Observational	Human	—	• Musculoskeletal health • Osteoporosis • Sarcopenia • Concentrations of serum inflammatory markers • Bone Loss • Incidence of fractures • Inflammatory status • Hyperlipidemia • LDL cholesterol • Rheumatoid arthritis • Autoimmune inflammatory diseases	• The dietary inflammatory index score was related to musculoskeletal health, such as osteoporosis and sarcopenia, among older adults >65 y. • A less inflammatory dietary pattern approach may decrease concentrations of serum inflammatory markers and, further, may prevent bone loss, sarcopenia, and the incidence of fractures. • Hyperlipidemia, concentrations of LDL cholesterol, and the use of statins should be adjusted in this study, as well as other autoimmune inflammatory diseases, such as SLE.	[53]
Observational	Human	—	• Prevalence of self-reported diagnosis/treatment of SLE • Dietary patterns • Smoking history	• Vegetarians had lower odds of doctor-diagnosed SLE with an increasing trend in prevalence from stricter vegetarians to pesco-vegetarians to nonvegetarians. • Ever smokers were more likely to report prevalent SLE than those who had never smoked (OR: 1.71, 95% CI: 1.27, 2.31).	[54]
Observational and cross-sectional	Human	—	• Plasma selenium concentrations • Erythrocyte glutathione peroxidase activity (GPx) • Plasma malondialdehyde (Mda) • Ultrasensitive C-reactive protein (UsCRP) • Insulin concentrations • Glycemia (HOMA-IR) • SLE Disease Activity (Measured By SLEDAI-2K) • Lipid	• Approximately 50% of adolescents with jSLE had below reference selenium concentrations. • GPx activity was more frequently below reference concentrations in the jSLE group compared with controls. • There was an independent inverse association between selenium and c-LDL concentrations in both groups.	[55]
Cross-sectional	Human	—	• Self-reported disease symptoms • General aspects of health • Symptom severity ratings • Weight loss • Fatigue • Joint/muscle pain • Mood	• Over 80% of patients with SLE who changed their eating patterns to include more plant-based foods and limit processed foods and animal products reported improvements in their disease symptoms. • The greatest decreases in symptom severity were provided by low/no dairy, low/no processed foods, and vegan eating patterns. • Weight loss, fatigue, joint/muscle pain, and mood were the most cited symptoms that improved with dietary change.	[56]
Cross-sectional	Human	—	• Dietary patterns • Lifestyle habits • Clinical features • Autoantibody spectrum	• Patients with SLE complicated with gastrointestinal involvement (SLE-GI) had higher proportions of vegetarians and lower proportions of omnivores than healthy controls. • Patient with SLE-GI had higher rates of taking traditional Chinese medicine and having a surgical history than healthy controls. • Patient with SLE-GI had lower frequencies of taking fried/pickled food and dietary supplements than healthy controls.	[57]
Cross-sectional	Human	—	• Hyperuricemia • Clinical activity • Renal activity • Triglycerides • Hs-CRP • Kannel score • BMI • HDL cholesterol	• Adequate protein and carbohydrate intake, healthy HDL cholesterol serum concentrations, and hydroxychloroquine treatment were associated with a lower risk of hyperuricemia in patients with SLE. • Patients with SLE with hyperuricemia presented a higher risk of clinical and renal activity, as well as worse cardiometabolic status. • Prednisone treatment was associated with a high risk of hyperuricemia.	[58]
Cross-sectional	Human	—	• Mediterranean Diet Adherence Score (MEDAS) • Physical activity energy expenditure (PAEE) • Smoking status • SLE disease activity (measured by SLEDAI-2K) • Depression scale (CES-D) • Fatigue severity (FSS) • Functional status (FFbH) • Physical and mental quality of life (PCS and MCS).	• The patients with SLE that followed a healthy lifestyle showed a higher physical quality of life, lower depression, lower fatigue, and lower dsDNA antibodies compared with their SLE counterparts that did not follow a healthy lifestyle regimen. • Physical activity had the highest impact on the various health domains when compared with smoking or diet adherence	[59]
*In vivo* trial	Mice	3 different commercial diets: Teklad 7013, Harlan 2018, or Research Diets Inc	• Proteinuria • Glomerular immune complex deposition • C3 • Total IgG • IgG1 • IgG3 • CD11B+ cellular infiltration into the glomeruli • Cytokine production • Fecal microbiota • MicroRNAs (miRNAs) • DNA methylation	• Diet alone can have an impact on immune complex glomerulonephritis, renal cellular infiltrates, microbiota and molecular behavior of cells after LPS activation	[60]
*In vivo* trial	Mice	Dietary resistant starch (RS)	• Percent survival • Weight (spleen and liver) • Relative expression: IFNA1, IFNB (spleen and ileum) • Systemic type 1 IFN • CD11c and PDCA-1 pDCs (CD45+ cells) • Glomerulonephritis (histopathologic scores) • Proteinuria • Fecal bacterial DNA sequenced with linear discriminant analysis effect size (LDA) score • Bacterial translocation • Translocation distribution • Relative abundance of bacteria • Frequencies of pDCs in spleen, MLN, SI-LP • Blood urea nitrogen	• Western lifestyle is linked to autoimmune and metabolic diseases, driven by changes in diet and gut microbiota composition. • *Lactobacillus reuteri* can drive autoimmunity but is ameliorated by dietary RS. • RS suppresses the abundance and translocation of *L. reuteri*, inhibiting its growth and decreasing pDCs, IFN pathways, organ involvement, and mortality in lupus-prone hosts.	[26]
*In vivo* trial	14 Organisms: cow, sheep, goat, pig, chicken, turkey, duck, tilapia, salmon, rice, quinoa, soybean, rye, wheat)		• Global Gershteyn-Ferreira (GF) index • Shared epitopes and Unique epitopes • Diseases with single species epitope matches (number of hits): alopecia aerata, antiphospholipid syndrome, autoimmune atherosclerosis, bullous pemphigoid, cutaneous lupus erythematosus, demyelinating polyneuropathy, Dermatomyositis, Goodpasture’s syndrome, Guillain-Barre syndrome, non-insulin dependent diabetes mellitus, Reactive arthritis, Rheumatic myocarditis, Vitiligo • Autoimmune unique GF index	• A total of 14 species investigated could be divided into 3 broad categories regarding their content in human autoimmune epitopes, which we represented using a new metric, the GF index. • Strikingly, pig contains a disproportionately high number of unique autoimmune epitopes compared with all other species analyzed. • This work uncovers a potential new link between pork consumption and autoimmunity in humans and lays the foundation for future studies on the impact of diet on the pathogenesis and progression of autoimmune disorders.	[61]
*In vivo* trial	Mice	HFD (high-fat diet)	• Body weight gain, Spleen weight • Flow cytometry: B220GLT7+CD38- GC B cell in splenocytes, effector memory CD4+ and CD8+ T cells, expression of CD3, CD4, CD8, CD44, and CD62L, Treg cells (CD4+Foxp3+) and their activation status (CD44+CD69+) • Serum anti-DNA and anti-RNA, and IgM autoantibodies • Kidney pathology • Liver inflammation • Expression concentration of TNF, IL-6, IL-1β mRNA, Foxp3, IL-10, TLR7 • Glucose tolerance test concentrations	• TLR7 signaling is implicated in HFD-induced metabolic syndrome and exacerbation of lupus autoimmunity in TLR8-deficient (TLR8ko) mice. • HFD led to an increase in TLR7 expression in WT mice that was coupled with increased TNF production by DCs, and this phenotype was more profound in TLR8ko mice. • TLR7 might be a novel approach as a tailored therapy in SLE and metabolic diseases.	[27]
*In vivo* trial	Mice	3 different diets (control, Western diet, caloric restriction)	• Phenotyping complex traits in AIL mice: CRP, ANA, NASH, NAFLD, Ballooning, Steatosis, biGal, Sial/gal, termGal, G2S2, G2S1, G1S1, G2, G1, G0, G/M, A/M, A/G, AG, IgM, IgG, IgA, MPV, PLT, RDW, MCHC, MCH, MCV, HCT, HB, RBC, BA%, EO%, MO%, LY%, NE%, BA, EO, MO, LY, NE, WBC, Spleen, LDL, HDL, Cholesterol, WT(6M), WT(4M), WT(2M), AG/M, AGM, Sial, Gal, MonoGal • Manhattan plot showing QTL (quantitative trait locus) in the AIL population associations with various complex traits • Schematic representation of fine-mapped genes for weight in chromosome 5 • Impact of diet and sex on body weight, Proteinuria, Crescent formation score, frequency of PAS+ depositions (kidney injury), and presence of ANA • Alpha diversity (Chao1 index and Shannon index species richness) of mice, alongside principal coordinate analysis of beta diversity of microbiota • Abundance of mycobiota and microbiota taxa • Fungal microbial (FMC) and fungal community (FFC) trait correlations between sex, diet, stage, and disease, and correlations between FMC s with FFCs • Differentially expressed genes: FFC4, FMC1, Diet, Disease, Tg, Irf7, Oas2, Zbp1, Sct, Ms4a6c, Gcnt2, Nr4a1, Dhx58, TIr7, Pik3r6, Mapkapk2, SIc39a1, Rtp4, Ifi44, Oas1g, Oas1a, CbIn1, Cys1, Tnxb, Gabrq, PId5, Stmn2, SIco2b1, PIekha6, Iqgap2, Cnn3, Mturn, Ighv1-31, Ighv1-18,22,25,26, OlfmI2a, SIc16a5, 9030619P08Rik, Mir7649, Gm15987 • Presence of ANA • Genes present in the ANA QTL on chromosome 17 • Differential expression of genes for the ANA phenotype and present within the ANA QTL: H2-Eb2, Myo1f, Tap1, Cfb, C2, H2-K1, Psmb9, Psmb8, Tnxb, Rps28, Col11a2	• Diet substantially contributes to the variability of complex traits and unmasks additional genetic susceptibility quantitative trait loci (QTL). • Whole-genome sequencing of the AIL founder strains resolves these QTLs to few or single candidate genes. • Diet modulates genetic susceptibility to lupus and shifts intestinal bacterial and fungal community composition. • With most negative outcomes for lupus being demonstrated with Western diet over caloric restriction of control diet.	[62]
*In vivo* trial	Mice	High-cholesterol diet	• Clinical features: body weight • Biochemical: total cholesterol, LDL, HDL, triglyceride, FFA, glycemia • Hematology: Hb, RBC, WBC, lymphocytes, PLT • Inflammation: MPO, TIMP-1, CXCL1 • Atherosclerotic lesion size, atherosclerotic • Macrophage and neutrophil infiltration • Collagen • MMP-8 and MMP-9 • Pro-MMP-9 expression • Anti-apoA-1 IgG concentrations • Anti-dsDNA IgG concentrations • Kidney size • Splenomegaly • Lymph nodes hypertrophy • mRNA concentrations of GATA3	• Apoe−/−Nba2.Yaa mice and Apoe−/− mice subject to a high-cholesterol diet had similar atherosclerosis lesion size in aortic roots and abdominal aorta. • Apoe−/−Nba2.Yaa mice showed higher concentrations of macrophage and neutrophil infiltration, collagen, MMP-8 and MMP-9 and pro-MMP-9 expression, indicating features of atherosclerotic plaque vulnerability. • Apoe−/−Nba2.Yaa mice had higher anti-apoA-1 and anti-dsDNA IgG concentrations, which correlated with mRNA concentrations of GATA3, IL-4, Bcl-6, and CD20 in the spleen and aortic arch.	[63]
[64]	Mice	High-fat diet (model for atherosclerosis)	• Lesion area • Serum cholesterol and LDL • CD4+, CD45+, CD25+, Monocytes, MHCII macrophages • Anti-dsDNA, anti-oxLDL IgG, anti-heart, anti-ApoH, anticardiolipin IgG, IgG2a • Spleen weight and cell count • Phenotypes of atherosclerosis • Differentiation and function of Treg cells • Proliferation index, CD25, Foxp3+, B6, Pbx1d, IL-21	• Pbx1d-transgenic T cells exacerbated some phenotypes of atherosclerosis in Ldlr-/- mice, which was associated with higher autoantibody production, increased Tfh cell frequency, and impaired Treg cell regulation. • Dyslipidemia and Pbx1d-transgenic expression independently impaired the differentiation and function of Treg cells *in vitro.* • The combination of Pbx1d expression in T cells and dyslipidemia exacerbates both atherosclerosis and autoimmunity, at least in part through a dysregulation of Treg cell homeostasis.	[65]
*In vivo* trial	Mice	Low fiber intake	• Mortality: Survival %, albustix score, proteinuria • Autoantibody production: Anti-dsDNA-IgG, anti-dsDNA-IgG1, anti-dsDNA-IgG2a • Immune dysregulation: O.D., lymphoproliferation, spleen weight, kidney leukocyte infiltration, CD45+ cells/live cells, CD4+ and CD8+ T cells, activated CD4+ and CD8+ T cells, IL-10+/CD4+ T cells, Treg cells, effector Treg cells, Tfh cells, IL-6, IL-1β, TNF-α • Disrupted intestinal homeostasis: body weight, gonadal WAT (white adipose tissue), feces quantity, food intake, energy intake, colon length, small intestinal length, intestinal permeability • Gene expression: Muc2, Reg3, ZO1, IL-18, IL-6, IL-1β, TNF-α • Serum concentration: Leptin, IL-1β, IFNγ, IL-23, IL-6, TNF-α, GM-CSF, MCP-1, IL-17A, IL-10, IL-27, IFN-β • SLEDAI with CRP, BMI, leptin, and LPS	• Low dietary fiber intake is linked to the development of obesity and lupus pathogenesis. • Short-chain fatty acids and gut microbiota are implicated in the mode of action of beneficial fiber effects. • Low fiber-fed mice showed an increase in WAT mass, fat-inflammation, and a disrupted intestinal homeostasis, leading to systemic, low-grade inflammation driving autoimmunity.	[66]
*In vivo* trial	Mice	Chow diet compared with a high-fat diet, and methylprednisolone treatment	• Percent survival • Percent proteinuria • Urine protein/creatinine • Anti-dsDNA antibodies • BUN • Intraperitoneal Glucose Tolerance Test (IPGTT) • Body composition: body weight, fat mass, lean mass • Flow cytometry: T cell, T helper cell subset, and macrophage proportions in the spleen, inflammatory cells in stromal vascular cells from epididymal WAT • Kidneys analysis: mesangial proliferation, inflammatory cell infiltration, tubular dilation, fibrosis, IgG deposition, C3 deposition • Serum cytokines: IFN-γ, IL-1β, IL-2, IL-4, IL-10, IL-12p70, IL-15, IL-17A, IP-10, IL-6, MCP-1, TNF-α, MIP1b • Serum insulin, leptin, PAI1, Resistin, adiponectin, FFA, glycerol, TC, TG	• Treatment with methylprednisolone significantly increased the survival in the control diet group, but not in the HFD group. An HFD significantly increased the incidence of severe proteinuria and glucose intolerance. • Treatment with methylprednisolone significantly lowered the serum concentrations of IL-6, MCP-1, and TNF-α in the control diet group, but not in the HFD group. • These data improve our understanding of the effect of HFD on the therapeutic efficacy of corticosteroids in SLE treatment, which could have clinical implications.	[67]
*In vivo* trial	Mice	High-dose methyl diet	• Anti-dsDNA IgG antibodies concentrations • Proteinuria concentrations • Skin lesion score • % of Survival • IFN-γ and IL4 secretion score • Glomerular score • DNA methylation concentrations	• Methyl-rich diet was found to decrease concentrations of proteinuria, anti-dsDNA antibodies, and modulate cytokine profiles in MRL/lpr mice. • Methyl-rich diet limited kidney failure and prevented the development of skin lesions in MRL/lpr mice.	[68]
*In vivo* trial	Mice	TLR7 agonist imiquimod (IMQ) and a high fat, high sucrose “Western diet” (HFD) intervention	• Spleen weights • Antinuclear antibody (ANA) positivity • Body Weight • Gonadal fat pad mass • Plasma leptin concentrations • FBG concentrations • Fasting insulin concentrations	• TLR7 agonist treatment combined with a high-fat, high-sucrose diet (HFD) affects the early-stage development of SLE or MetS features. • TLR7 agonist treatment and HFD exposure increased body weight, gonadal fat pad mass, and plasma leptin concentrations. • TLR7 agonist treatment affected fasting insulin concentrations in a diet-dependent manner, resulting in hyperinsulinemia in IMQ-HFD-treated mice.	[69]
*In vivo* trial	Mice	Iron supplementation	• Tfh cell expansion • Proinflammatory cytokine secretion • Autoantibody production • Antigen specific Gc response	• Iron overload promotes Tfh cell expansion, proinflammatory cytokine secretion, and autoantibody production in lupus-prone mice. • Iron supplementation contributes to Tfh cell differentiation, whereas iron chelation inhibits Tfh cell differentiation. • The miR-21/BDH2 axis drives iron accumulation during Tfh cell differentiation and further promotes Fe2+-dependent ten-eleven translocation (TET) enzyme activity and BCL6 gene demethylation.	[70]
*In vivo* trial	Mice	High-fat diet (HFD)	• Anti-DsDNA • Proteinuria • Increased creatinine • Fluorescein isothiocyanate dextran • Serum LPS • Serum IL-6 • Alanine transaminase (ALT) • Liver and kidney pathology • Activated caspase 3 • Aorta thickness	• Obesity (induced by HFD) exacerbates lupus activity in FcgRIIb–/– lupus mice, partly through SFA-induced gut barrier defect and systemic inflammation. • High-fat diet administration in FcgRIIb–/– mice resulted in increased lupus nephritis, gut barrier defect, serum LPS, serum IL-6, liver injury, organ fibrosis, spleen apoptosis, and aorta thickness. • Combined palmitic acid and LPS induced higher TNF-α, IL-6, and IL-10 in FcgRIIb–/– macrophages compared with wild-type macrophages.	[71]
*In vivo* trial	Mice	HFD	• Bregs and their regulatory effects • Th17/Treg cell balance • Release of downstream inflammatory factors	• CD19+CD5+CD1d+ Bregs are present in mice with SLE complicated with atherosclerosis. • Bregs regulate the Th17/Treg balance and the release of downstream inflammatory factors. • Bregs may play a role in maintaining the Th17/Treg balance in mice with SLE complicated with atherosclerosis.	[72]
*In vitro* trial	Human	Low or high-dose folate coculturing	• Secretion of IL-10 from regulatory cells • Methylation concentration of B lymphocytes	• Folic acid coculturing with PBMCs from patient with lupus and healthy volunteers resulted in increased IL-10 secretion from regulatory B lymphocytes. • Low-dose folic acid coculturing had a greater effect on IL-10 secretion than high-dose folic acid coculturing. • Folic acid coculturing had no effect on IL-10 secretion from non-regulatory B lymphocytes.	[73]
Systematic review	Human	—	• Pain • Disability • Outcomes of people with RMDs • Diet • Exercise • Weight loss • Smoking • Working	• Five overarching principles and 18 specific recommendations were developed based on available evidence. 1. OP 1: Lifestyle improvements complement medical treatment and do not replace it 2. OP 2: Lifestyle improvements are an essential part of RMD management and add to overall health benefits 3. OP 3: WHO recommendations for a healthy lifestyle are also applicable to people with RMDs 4. OP 4: Lifestyle recommendations for individuals with RMD depend on factors such as age, sex, health condition, pregnancy, and comorbidities 5. OP 5: There should be regular discussions between people with RMDs and health professionals regarding lifestyle factors • Recommendations emphasize the importance of a healthy lifestyle, how lifestyle modifications should be implemented, and their role in relation to medical treatments.	[74]

Abbreviations: ACPA, anticitrullinated protein antibody; AIL, advanced intercross line; APS, antiphospholipid syndrome; ARDs, autoimmune rheumatic diseases; BA, basophils; Bregs, regulatory B cells; BUN, blood urea nitrogen; CI, confidence interval; CLE, cutaneous lupus erythematosus; CD, Celiac disease; CNS, central nervous system; CVD, cardiovascular disease; CXCL1, C-X-C motif chemokine ligand 1; DAS28, Disease Activity Score-28; DMA, dimethylamine; EO, eosinophils; ESRF, end-stage renal failure; FFA, free fatty acid; FFC, fungal fungal community/features; FMC, fungal microbial community/features; GATA3, GATA-binding protein 3; HCT, hematocrit; HCQ, hydroxychloroquine; IMQ, imiquimod; JDM, juvenile dermatomyositis; JIA, juvenile idiopathic arthritis; jSLE, juvenile systemic lupus erythematosus; LY, lymphocytes; MCH, mean corpuscular hemoglobin; MCHC, mean corpuscular hemoglobin concentration; MCTD, mixed connective tissue disease; MCV, mean corpuscular volume; MetS, metabolic syndrome; MLN, mesenteric lymph nodes; MMF/MPA, mycophenolate mofetil/mycophenolic acid; MO, monocytes; MPO, myeloperoxidase; MPV, mean platelet volume; NAFLD, nonalcoholic fatty liver disease; NASH, nonalcoholic steatohepatitis; NE, neutrophils; OP, overarching principle; OR, odds ratio; PAS, periodic acid–Schiff; PBMCs, peripheral blood mononuclear cells; p-CS, p-cresol sulfate; pDCs, plasmacytoid dendritic cells; QTL, quantitative trait locus; RA, rheumatoid arthritis; RDW, red cell distribution width; RF, rheumatoid factor; RMDs, rheumatic and musculoskeletal diseases; SI-LP, small intestine lamina propria; SLICC, Systemic Lupus International Collaborating Clinics ; Tfh, T follicular helper; TIMP-1, tissue inhibitor of metalloproteinase-1; WT, wild type.

### Essential fatty acids

Essential fatty acids, specifically omega (ω)-3 and ω-6 fatty acids, are critical components of the human diet, as they cannot be synthesized by the body. These PUFAs play significant roles in modulating immune responses, which has drawn substantial attention for their influence on the pathogenesis and progression of autoimmune diseases, including SLE [75,76]. ω-3 fatty acids, such as EPA and DHA, are metabolized into eicosanoids, lipid-based signaling molecules that participate in immune responses. Unlike eicosanoids derived from ω-6 fatty acids, those from ω-3 are generally less inflammatory, contributing to a more balanced immune response [77]. Moreover, EPA and DHA are precursors to specialized proresolving mediators such as resolvins, protectins, and maresins, which actively resolve inflammation, promote tissue repair, and aid in microbial clearance [78].

On the other hand, ω-6 fatty acids, with linoleic acid (LA) as a primary example, are converted into arachidonic acid, which serves as a precursor for eicosanoids like prostaglandins, thromboxanes, and leukotrienes [76]. These eicosanoids are typically proinflammatory and play essential roles in initiating and promoting inflammatory responses necessary for combating infections and repairing tissue damage [79,80]. However, when inflammation becomes chronic, these proinflammatory eicosanoids can contribute to autoimmune disease development. The balance between ω-3 and ω-6 fatty acids is therefore crucial for maintaining immune homeostasis [81]. Research has shown that this balance is particularly important in autoimmune conditions such as lupus, where dietary interventions involving omega fatty acids have demonstrated potential benefits in disease management [82,83].

Several clinical studies have explored the impact of ω-3 supplementation in patients with SLE. Arriens et al. [84] conducted a randomized placebo-controlled trial in which 50 patients with SLE received daily fish oil supplementation containing 2.25 g of EPA and 2.25 g of DHA for 6 mo. The ω-3 supplemented group showed significant improvements in quality of life, as assessed by the RAND Corporation 36-Item Short Form Health Survey (RAND SF-36), and reductions in disease severity, based on the Physician Global Assessment. Additionally, circulating inflammatory markers were reduced in the treatment group [84]. Similarly, Borges et al. [85] found that after 12 wk of daily supplementation with 1080 mg EPA and 200 mg DHA, patients with SLE exhibited a reduction in C-reactive protein (CRP), an inflammatory marker, though no significant changes were observed in IL-6, IL-10, leptin, or adiponectin concentrations.

Further studies have examined the relationship between PUFA profiles and inflammation in patients with SLE. Vordenbäumen et al. [83] conducted a cross-sectional study of the erythrocyte membrane PUFA profiles of 68 adult patients with SLE. They observed that higher concentrations of ω-6 PUFAs and a higher ratio of LA to ALA were associated with increased CRP concentrations. Conversely, a higher percentage of ω-3 PUFAs was inversely correlated with CRP concentrations, indicating a potential anti-inflammatory effect of ω-3s. Moreover, increased intake of ω-3-rich foods, such as fish, was associated with an improved ω-3 status and a reduction in self-reported disease damage, as measured by the Brief Index of Lupus Damage [83]. Similar findings were reported by Charoenwoodhipong et al. [82] in a cohort of 456 patients with SLE from the Michigan Lupus Epidemiology and Surveillance cohort. They found that for every unit increase in the ω-6 ratio, the Systemic Lupus Activity Questionnaire (SLAQ) score increased by 0.3 points (higher SLAQ scores indicate greater disease activity and symptom severity). Additionally, each gram increase in ω-3 PUFA consumption per 1000 kcal was associated with a decrease in lupus activity and fewer sleep disturbances, as measured by the Patient-Reported Outcomes Measurement Information System [82].

These findings collectively highlight the importance of the ω-6 to ω-3 ratio in modulating inflammation and disease outcomes in SLE. A higher ratio of ω-6 to ω-3 PUFAs appears to correlate with increased inflammation and disease activity, whereas increasing ω-3 intake is associated with improved clinical outcomes and reduced inflammatory markers. Thus, balancing ω-3 and ω-6 fatty acids through dietary modifications may be a valuable strategy in managing autoimmune diseases like lupus.

### Animal studies suggest modes of action behind the potential protective effect of ω-3 PUFAs in lupus

In lupus mouse models induced by crystalline silica (cSiO_2_), supplementation with DHA has been shown to significantly inhibit the proliferation of immune cells such as B cells, T cells, follicular dendritic cells, and IgG-positive plasma cells within the lungs [86]. This supplementation also reduced concentrations of anti-dsDNA IgG in both bronchial lavage fluid and plasma. Additionally, DHA attenuated the development of glomerulonephritis, alongside a reduction in B-cell accumulation in the renal cortex [86]. Furthermore, a diet high in DHA mitigated the upregulation of genes associated with inflammation, immune responses (both innate and adaptive), interferon (IFN) signaling, chemokines, and antigen processing in lupus models [87]. Dietary DHA intake in lupus-prone mice also inhibited the expression of Mrna signatures commonly linked to the formation of ectopic lymphoid tissues, systemic autoimmunity, and glomerulonephritis [87].

In the lupus mouse model induced by cSiO_2_, there was a marked autoantibody response, particularly IgG, IgM, and to a lesser extent, IgA. However, DHA supplementation dose dependently reduced these autoantibody concentrations, which were inversely correlated with ω-3 fatty acid concentrations in tissue phospholipids [88]. This negative correlation extended to the activation of IFN-regulated genes, production of proinflammatory cytokines, leukocyte infiltration, ectopic lymphoid structure formation in the lungs, systemic autoantibody production, and glomerulonephritis development [89].

Additionally, Pestka et al. [90] compared the effects of ω-3, ω-6, and ω-9-rich diets in lupus-prone mice. Mice fed ω-6 or ω-9 diets exhibited elevated concentrations of plasma autoantibodies, proteinuria, and glomerulonephritis. In contrast, mice on an ω-3-rich diet showed significantly lower levels of these symptoms. This suppression of autoimmune responses by ω-3 was linked to the downregulation of CD4+ T cell-related genes, including CD80, CTLA-4, IL-10, IL-18, CCL-5, CXCR3, IL-6, TNF-α, and osteopontin in kidney and spleen tissues, relative to the ω-6 and ω-9 diets [90]. These genes are involved in inflammatory responses, antigen presentation, T-cell activation, B-cell activation and differentiation, and leukocyte recruitment.

Table 2 [[82], [83], [84], [85], [86], [87], [88], [89], [90], [91], [92], [93], [94], [95], [96], [97]] provides a summary of the relationships between essential fatty acids and the incidence and severity of lupus, highlighting the potential therapeutic role of ω-3 in modulating immune and inflammatory responses in lupus-prone models.

**TABLE 2:** The relationships between essential fatty acids and lupus incidence and severity

Detailed study design	Organism	Interventions/nutrient	Outcome measured	Study result	Reference
Observational	Human	—	• Demographic: age, gender, race/ethnicity, BMI, fat energy intake, dietary n–3 and n–6 intake, flaxseed/fish oil supplementation • Patient-reported outcomes in systemic lupus erythematosus (SLE): Systemic Lupus Activity Questionnaire (SLAQ), Survey Criteria for Fibromyalgia (FM Scale), Patient-Reported Outcomes Measurement Information System (PROMIS), RAND Medical Outcomes Study Short Form 36 (SF-36), LupusQoL domains	• Dietary intake of ω-3 and ω-6 fatty acids were associated with patient-reported outcomes in SLE. • Higher intake of ω-3 fatty acids were associated with lower levels of fatigue, pain, and depression. • Higher intake of ω-6 fatty acids were associated with higher levels of fatigue, pain, and depression.	[82]
Cross-sectional	Human	—	• Patient characteristics: gender, disease duration, BMI, SLAQ, Brief Index of Lupus Damage (BILD), CRP, C3c, Medications • Distribution of PUFAs: arachidonic acid (AA), linoleic acid (LA), EPA, DHA, α-linoleic acid (ALA), ω-6%, ω-3%, δ-5 desaturase index, δ-6 desaturase index • Associations of PUFA and clinical parameters in patients with SLE: ω-6% and ω 3% in HUFA with CRP concentrations, ω-3 salute and BILD Score with PUFA fish intake	• ω-6 PUFAs were associated with higher concentrations of systemic inflammation (measured by CRP). • ω-3 PUFAs were associated with lower concentrations of systemic inflammation. • Increased dietary PUFA consumption from fish was linked to higher ω-3 status and lower self-reported damage.	[83]
Randomized controlled trial	Human	Fish oil (EPA and DHA)	• Physician Global Assessment (PGA) • RAND Short Form-36 (Rand SF-36) • Fatigue Severity Scale (FSS) • SLE Disease Activity Index (SLEDAI) • Serum IL-12 • Serum IL-13 • Erythrocyte Sedimentation Rate (ESR)	• Fish oil supplementation improved PGA compared with placebo (*P* = 0.015). • Trends of improvement were seen in RAND SF-36 Energy/fatigue and Emotional well-being scores (*P* = 0.092 and 0.070). • Erythrocyte sedimentation rate and serum IL-12 were reduced (*P* = 0.008 and 0.058), whereas serum IL-13 was increased by fish oil supplementation (*P* = 0.033).	[84]
Clinical trial	Human	Supplementation with ω-3	• Circulating concentrations of inflammatory mediators • Biochemical markers • CRP concentrations • Serum concentrations of IL-6 • Serum concentrations of IL-10 • Leptin concentrations • Adiponectin concentrations • Total cholesterol concentrations (TC) • LDL cholesterol concentrations (LDL)	• ω-3 supplementation in females with low-activity SLE didn't affect IL-6, IL-10, leptin, and adiponectin concentrations but significantly reduced CRP concentrations and potentially affected total and LDL cholesterol.	[85]
*In vivo* trial	Mice	1) n–3 PUFA-rich diet containing DHA-enriched fish oil, 2) n–6 PUFA-rich Western-type diet containing corn oil or 3) n–9 MUFA-rich Mediterranean-type diet containing high oleic safflower oil	• Plasma autoantibodies • Proteinuria • Glomerulonephritis • Expression of 84 genes associated with CD4+ T-cell function in the spleen and kidney • CD80 mRNA expression in kidney and/or spleens • CTLA-4 mRNA expression in kidney and/or spleens • IL-10 mRNA expression in kidney and/or spleens • IL-18 mRNA expression in kidney and/or spleens	• Mice fed a n–3 PUFA-rich diet containing DHA-enriched fish oil (DFO) had significantly reduced plasma autoantibodies, proteinuria and glomerulonephritis compared with mice fed n–6 PUFA or n–9 MUFA diets. • Consumption of the *n*-3 PUFA diet was associated with a generalized downregulation of CD4+ T cell-related genes in kidney and/or spleen at week 34. • Quantitative RT-PCR of representative affected genes confirmed that n–3 PUFA consumption was associated with reduced expression of CD80, CTLA-4, IL-10, IL-18, CCL-5, CXCR3, IL-6, TNF-α, and osteopontin mRNAs in kidney and/or spleens as compared with mice fed n–6 PUFA or n–9 MUFA diets. • These genes are associated with the inflammatory response, antigen presentation, T-cell activation, B cell activation/differentiation, and leukocyte recruitment in kidney and/or spleens.	[90]
*In vivo* trial	Mice	DHA	• Inflammatory and autoimmunity markers in lung, blood, and kidney	• DHA suppressed lung inflammation. • DHA reduced serum concentrations of proinflammatory cytokines, antibodies, and B-cell proliferation. • DHA reduced glomerulonephritis in kidney.	[91]
*In vivo* trial	Mice	DHA	• Pulmonary ectopic germinal center Formation. • Glomerulonephritis. • Inflammation in lungs • B cell and T-cell accumulation in lungs • IgG+ plasma cell appearance in lungs	• Dietary DHA prevents silica-induced development of pulmonary ectopic germinal centers, glomerulonephritis, as well as increased B cell, T cell, follicular dendritic cell (FDC), and IgG+ plasma cell appearance in the lungs of lupus-prone NZBWF1 mouse	[86]
*In vivo* trial	Mice	DHA	• Acute transcriptional response of immune-associated genes in DHA-supplemented mice • PCA plot of differentially expressed genes (PC1 and PC2) • Global and directed significance scores for immune pathways: adaptive, adhesion, antigen processing, apoptosis, B-cell functions, CD molecules, cancer progression, cell cycle, chemokines and receptors, complement pathway, cytokines and receptors, dendritic cell functions, humoral, inflammation, innate, IFN, ILs, leukocyte functions, MHC, macrophage functions, microglial functions, NK cell functions, pathogen response, senescence, T-cell functions, TLR, TNF superfamily, transporter functions • Pathway Z scores and network interaction visualization: adaptive, antigen processing, CD molecules, chemokines and receptors, inflammation, innate, IFN, IL, MHC, macrophage functions, T-cell functions • Unsupervised clustering by gene depicts log2 expression ratio: innate, adaptive inflammation, chemokines and receptors, IFN • Log2 ratio values: Ccl7, Ccl12, Cxcl10, Fcer2a, Fcgr1, Ifi44, Ifit1, Ifit3, Ifrf7, Isg15, Klrg1, Mx2, Oas2, Ppbp, Zbp1, Nlrc5, Irgm2, Clec4a2, Cfd, Mx1, Mx2, Marco, Cxcl9, Ccl8, Cxcl13, Ccr6, Stat1, C3ar1, Tlr1, Cd180, Ly86, Il1b, Fos, Cxcl5, Ccl17, Ccl20, Ccr5, Fos, Clec4a2, Lcn2, S100a8, Ccl8, Cd5, Il1b, Il1m, Elane, Tlr8, Tlr4, Ly86, Cx3cr1, Cxcl14, Ccl24, Fcer1g, Ccl24, H2-Dmb2, H2-Eb1, Icam1, Sh2d1b1, Nlrc5, gene • Percent ω-3 HUFA in erythrocytes (ω-3 HUFA score)	• Dietary supplementation with DHA suppresses autoimmune pathogenesis and nephritis in lupus-prone female NZBWF1 mice. • DHA consumption interferes with upregulation of critical genes associated with cSiO2-triggered murine lupus. • DHA dose-dependently suppresses interferon (IFN)- and chemokine-related gene pathways in response to cSiO2 treatment.	[87]
*In vivo* trial	Mice	Dietary ω-3 PUFA DHA	• IgG AAb responses in BALF and plasma • IgM AAb responses in BALF and plasma • IgA AAb in BALF and plasma • Ectopic lymphoid structure (ELS) development • Glomerulonephritis	• Crystalline silica (cSiO_2_) exposure induces a wide spectrum of autoantibodies (AAbs) in the pulmonary and systemic compartments of lupus-prone NZBWF1 mice. • cSiO2 triggers robust IgG and IgM AAb responses against lupus-associated antigens, including DNA, histones, ribonucleoprotein, Smith antigen, Ro/SSA, La/SSB, and complement. • Dietary DHA intervention prevents cSiO_2_-induced inflammation and onset of autoimmunity (suppressed increases in IgG and IgM AAbs in BALF and plasma).	[92]
*In vivo* trial	Mice	ω-3 HUFAs EPA and DHA	• Indicators of autoimmune pathogenesis in the cSiO_2_-triggered lupus flaring model • Expression of IFN-regulated genes • Proinflammatory cytokine production • Leukocyte infiltration • Ectopic lymphoid structure development in the lung • Pulmonary and systemic autoantibody production • Glomerulonephritis • ω-3 HUFA score and Omega-3 Index (O3I)	• Increases in both the ω-3 HUFA score (>40%) and the O3I (>10%) were strongly associated with suppression of cSiO_2_-triggered autoimmune pathogenesis in the lung, autoantibody production, and glomerulonephritis. • The ω-3 HUFA scores in RBCs were comparatively more robust than the O3I at predicting HUFA balances in the kidney, liver, spleen, and lung. • These findings identify achievable ω-3 HUFA scores and O3I thresholds that could be targeted in future human intervention studies querying how ω-3 HUFA consumption influences lupus and other autoimmune diseases.	[89]
*In vivo* trial	Mice	Western-fed diet, and group supplemented with dietary ω-3 PUFA DHA	• Weight gain • % Total fatty acids: RBC, lung, kidney, liver, spleen • IFN Score comprised: Ccl7, Zbp1, Ifi44, Ifit1, Irf7, Isg15, Mx1, Oas1, Oas2, Oasl1, Psmb8, Rsad2, Siglec1, Ccl8, Cxcl10 • Cytokine concentrations: MCP-1, MCP-3, TNF-α, IL-1α, IL-6, IL-18, IL-17A, IL-22, BAFF • Immune cell accumulation in BALF: total cells, monocytes, lymphocytes, neutrophils, lymphocytes • CD45R+ and CD3+ cells in the lungs • Autoantibodies in BALF and plasma: anti-dsDNA and antinuclear antibody (ANA) • ELS neogenesis • Histology score (glomerulonephritis)	• Consumption of a modified Total Western Diet (mTWD) containing DHA at the caloric equivalent to a human dose of 5 g/d dramatically suppressed induction of all lupus-associated endpoints in NZBWF1 mice exposed to crystalline silica (cSiO_2_). • Decreasing SFA and ω-6 in mTWD modestly inhibited some disease markers, but DHA addition to this diet was required for maximal protection against lupus development. • DHA supplementation at a translationally relevant dose was highly effective in preventing cSiO_2_-triggered lupus flaring in NZBWF1 mice.	[93]
*In vivo* trial	Mice	Dietary supplementation with EPA	• Autoantibody production: ANA, anti-dsDNA, antihistone, IgG, C3, IgM, BAFF • IFN-α/β, White Pulp (WP) area • Serum fatty acid, TG, cholesterol • Spleen plasma cell count: total CD4+, Naïve, Teff (T effector cells), Treg (T regulatory cells), Tfh (T follicular helper cells), total B, FOB, MXM, GCB, plasma, population, cell count, DNT (double negative T cells), total B, FOB (follicular B cells), MZB (marginal zone B cells), GCB (germinal center B cells) • Inflammatory cytokines: IL-12p40, IL-6, TNF-α, IFN-β. IL-12p70, IFN-α/β • Gene expression: Baff, Veh, LPS, R848, Cd79b, Prdm1, Xbp1, Irf4m • Blimp1 (mean fluorescence intensity) • Total and free fatty acids, free cholesterol, amount of PC phosphatidylcholine (PC) and PE (phosphatidylethanolamine) species	• Dietary supplementation with EPA inhibits plasma cell differentiation and attenuates lupus autoimmunity. • EPA remodels the lipid composition and fluidity of B cell membranes, preventing B cell differentiation into autoantibody-producing plasma cells. • EPA supplementation may be beneficial for therapy of lupus.	[94]
*In vivo* trial	Mice	DHA	• Body weight • % Total fatty acids: DHA-EPA and ARA concentration in kidney, lung, and plasma • Inflammatory cell recruitment: total leukocytes, macrophages, neutrophils, lymphocytes • ELS score • CD45R+ T-cell infiltration, CD21/35+ follicular dendritic cell infiltration • IgG antibodies • Selected autoantigens: C3, C1q, SP100, PR-3, LC1, Nup62, MI-2, vimentin • Nephritis Score (Glomerulonephritis) • Kidney CD3+ T-cell infiltration • Percent proteinuria • Percent survivors	• Dietary supplementation with DHA beginning 2 wk before cSiO_2_ challenge prevented inflammation and autoimmune flaring in NZBWF1 mice. • Dietary intervention with high but not low DHA after cSiO_2_ treatment suppressed or delayed recruitment of T cells and B cells to the lung, development of pulmonary ELS, and elevation of a wide spectrum of plasma autoantibodies associated with lupus. • DHA consumption dose-dependently increased ω-3 PUFA content in the plasma, lung, and kidney at the expense of the ω-6 PUFA AA.	[95]
*In vivo* trial	Mice	Dietary supplementation with the ω-3 PUFA DHA	• Inflammatory proteins in BALF • Inflammatory proteins in plasma	• Intranasal instillation of lupus-prone mice with crystalline silica (cSiO_2_) induces inflammatory gene expression and ectopic lymphoid neogenesis in the lung. • Dietary supplementation with the ω-3 PUFA DHA suppresses cSiO_2_-induced inflammatory proteins in bronchoalveolar alveolar lavage fluid (BALF) and plasma of lupus-prone mice. • High-density multiplex array profiling of 200 inflammatory proteins revealed that DHA supplementation blocked or delayed the induction of chemokines, enzymes, adhesion molecules, costimulatory molecules, TNF superfamily proteins, growth factors, and signal transduction proteins.	[96]
*In vivo* trial	Mice	ω-3 PUFA supplementation and soluble epoxide hydrolase (sEH) inhibition	• Blood urea nitrogen (BUN) • Proteinuria • Hematuria • Kidney histopathology • Spleen enlargement • Lymphoid hyperplasia • Inflammatory cell recruitment in the liver • Blood fatty acid profiles • Epoxy fatty acid concentrations • Glomerular IgG deposition • Plasma antibody responses	• Subchronic intraperitoneal injection of rough LPS (R-LPS) induced robust glomerulonephritis in NZBWF1 mice, whereas smooth LPS (S-LPS) and saline vehicle (VEH) did not. • Dietary supplementation with ω-3 DHA and/or the soluble epoxide hydrolase (sEH) inhibitor 1-(4-trifluoro-methoxy-phenyl)-3-(1-propionylpiperidin-4-yl) (TPPU)suppressed R-LPS-induced glomerulonephritis. • Lipidome modulation by dietary ω-3 PUFA supplementation or sEH inhibition suppressed R-LPS-accelerated glomerulonephritis in lupus-prone mice.	[97]

Abbreviations: AAbs, autoantibodies; ALA, α-linolenic acid; ARA, arachidonic acid; BALF, bronchoalveolar lavage fluid; BILD, Brief Index of Lupus Damage; C3c, complement component 3c; CCL-5, C-C motif chemokine ligand 5; CTLA-4, cytotoxic T-lymphocyte-associated protein 4; DFO, docosahexaenoic acid-enriched fish oil; FDC, follicular dendritic cell; GCB, germinal center B cells; HUFA, highly unsaturated fatty acid; LA, linoleic acid; Lcn2, lipocalin-2; MHC, major histocompatibility complex; O3I, omega-3 index; PC1, principal component 1; PC2, principal component 2; PCA, principal component analysis; PROMIS, Patient-Reported Outcomes Measurement Information System; RAND, RAND Corporation; RBC, red blood cells; SLICC, Systemic Lupus International Collaborating Clinics; SLAQ, Systemic Lupus Activity Questionnaire; WP, white pulp.

### Alcohol consumption

Moderate alcohol consumption has been observed to have a protective effect against lupus. In a study by Barbhaiya et al. [98], 125 new cases of SLE were identified in the NHS and 119 in the NHSII cohort. When comparing individuals who consumed alcohol to those who did not, a meta-analyzed multivariable hazard ratio (HR) for a cumulative alcohol consumption of ≥5 g/d was found to be 0.61 [95% confidence interval (CI): 0.41, 0.89], indicating a reduced risk of SLE. Notably, the protective effect was still evident when alcohol consumption occurred >4 y before diagnosis, with a similar HR of 0.61 (95% CI: 0.41, 0.91). Females who regularly consumed wine, in particular, demonstrated a significant reduction in SLE risk compared with nondrinkers, suggesting a potential correlation between moderate alcohol intake (≥5 g or ∼0.5 standard drinks per day) and a reduced risk of SLE [98]. This finding raises the possibility that polyphenols in wine, rather than alcohol alone, may play a crucial role in this protective effect.

Further insights into the relationship between alcohol consumption and SLE were provided by Hahn et al. [99], who discovered that stem cell factor (SCF) amounts decreased significantly with each gram of cumulative alcohol intake per day. Females consuming >5 g of alcohol daily exhibited SCF amounts that were 7% lower than those of nondrinkers. Although no significant associations were found between alcohol intake and other cytokines, the reduction in SCF amounts offers a potential mechanism through which alcohol could reduce the risk of SLE. Interestingly, this effect was independent of autoantibody status, further supporting the idea that alcohol, particularly when consumed in moderation, may play a role in reducing the incidence of lupus by lowering circulating SCF amounts [99].

An inverse relationship between alcohol intake and SLE risk is biologically plausible. Previous research has shown that alcohol can diminish cellular responses to immunogens and reduce the production of proinflammatory cytokines, including TNF, IL-8, and IL-6, in immune cells such as alveolar macrophages and peripheral blood monocytes [100]. In addition, wine contains polyphenolic compounds such as resveratrol, which have demonstrated antioxidant and anti-inflammatory properties [101], including modulation of IFN-γ [102] and reductions in serum concentrations of IL-1β and IL-18 in animal models [103], although the relevance of these findings to typical dietary intake in humans remains uncertain.

However, the potential benefits of moderate alcohol intake should be viewed in the context of its established health risks, including liver toxicity and increased risk of certain cancers [104]. Furthermore, J- or U-shaped associations between alcohol intake and adverse health outcomes, including rheumatoid arthritis (RA), cardiovascular diseases, and all-cause mortality have been reported [[105], [106], [107]]. Moderate alcohol intake (5–9.9 g/d) has been associated with the lowest concentrations of inflammatory biomarkers among females with preclinical RA [108]. Notably, Barbhaiya et al. [98] reported that the inverse association between alcohol intake and SLE risk became slightly stronger after excluding heavy drinkers (individuals consuming >30 g/d), suggesting a similar nonlinear relationship.

Table 3 [98,99,109] summarizes the potential impact of alcohol consumption on lupus incidence and disease severity, highlighting the nuanced role that moderate alcohol intake, particularly wine, may have in influencing SLE risk.

**TABLE 3:** The effect of alcohol consumption on lupus incidence and severity

Detailed study design	Organism	Interventions/nutrient	Outcome measured	Study result	Reference
Observational cohort	Human	Daily mean intake of 49 nutrients (e.g., vitamins, alcohol, fatty acids, etc.).	• Glucocorticoid use over 2-y period as a proxy for SLE disease activity.	• Linoleic acid (n–6 PUFA), β-carotene, and vitamin B-6 were inversely associated with unchanged/increased glucocorticoid dose. • Total energy intake was associated with higher glucocorticoid dose. • Alcohol was inversely associated with glucocorticoid treatment.	[109]
Observational cohort	Human	—	• Alcohol consumption • Risk of SLE	• An inverse association between moderate alcohol consumption (≥5 g or 0.5 drink/d) and SLE risk in females.	[98]
Cohort	Human	—	• Participant characteristics: age, smoking, alcohol intake, income, BMI, race, residence region, contraceptive use, menopause, steroid use • Cytokine/chemokine concentration: stem cell factor (SCF), interferon(IFN) inducible protein-10 (IP-10), IFN-α, B lymphocyte stimulator (BLyS), IL-10 • Antinuclear autoantibodies • Double-stranded DNA (dsDNA) • Extractable nuclear antigens (ENA)	• Moderate alcohol consumption was associated with lower stem cell factor amounts in female nurses without SLE. • Other cytokines were not significantly associated with alcohol intake. • “Moderate alcohol consumption was associated with lower SCF amounts, suggesting a plausible mechanism through which alcohol may lower SLE risk might be by decreasing circulating SCF.”	[99]

Abbreviation: SLE, systemic lupus erythematosus.

### Sodium intake

High-sodium intake worsens lupus symptoms. Animal studies show that a high-sodium diet (HSD) speeds up disease progression and makes lupus nephritis more severe, reducing survival inMurphy Roths large/lymphoproliferation (MRL/lpr) mice. This is linked to increased Th1 and Th17 immune cells. Using SGK1 (glucocorticoid-inducible serine/threonine protein kinase 1) inhibitors helped reduce these harmful effects. [110]. Moreover, HSD was shown to enhance the maturation and activation of bone marrow-derived dendritic cells through the p38 MAPK-STAT1 pathway, both *in vitro* and *in vivo* [111]. Another study inNew Zealand Black/New Zealand White F1 (NZBWF1) mice found that a HSD increased anti-dsDNA antibodies but did not change blood pressure or kidney damage. Urinary ET-1 increased, whereas some kidney markers (renal endothelin A receptor and IL-2) decreased, suggesting that long-term high sodium may not worsen heart and kidney problems in SLE [112].

Conversely, a clinical trial examining the effects of a low-sodium diet on patients with SLE and RA found that reducing sodium intake lowered proinflammatory Th17 cell counts, increased Treg cell counts, and decreased serum IL-9 concentrations. These effects were reversible upon returning to a regular sodium diet [113]. This finding aligns with earlier animal studies that suggested HSDs amplify systemic inflammation, whereas low-sodium diets mitigate it, particularly by affecting cytokine production and inflammation [110]. Notably, no significant changes in apoptotic human peripheral blood mononuclear cells (PBMCs) or factors modulating proliferation were observed during the trial, indicating that the diet specifically influenced inflammatory pathways without altering cell survival [113].

In a cross-sectional study on the Mexican-Mestizo population, a link was found between excessive weight (BMI >25) and sodium consumption [114]. Overweight individuals showed significant reductions in several micronutrients and macronutrients, hinting at poor dietary choices. Patients with overweight and SLE consuming fewer calories than their normal-weight counterparts could benefit from reduced sodium intake, but may also suffer from deficiencies in essential micronutrients that regulate immune function, such as vitamins B and C, zinc, and selenium [114]. Additionally, these patients frequently had low-quality diets, as indicated by their dietary antioxidant quality scores and elevated serum hsCRP concentrations, although these metrics were not significantly correlated [22]. Instead, the mineral content of their diets, particularly sodium and potassium concentrations, appeared to influence hsCRP concentrations in patients with SLE. High-sodium or low-potassium intake was linked to increased inflammation [22]. Furthermore, sodium intake was associated with elevated anti-dsDNA concentrations and reduced C4 complement protein concentrations, whereas potassium intake correlated with C3 complement protein concentrations [115]. However, another observational study found that higher sodium intake was inversely related to C3 and C4 concentrations in inactive patients with SLE. In this study, gut health was also explored, revealing a rise in *Megamonas funiformis* and plasma zonulin, both indicators of increased gut permeability [116].

Sodium intake influences both innate and adaptive immunity. In the innate immune system, prolonged HSDs are thought to promote inflammation, driven mainly by macrophages and dendritic cells. Although this heightened inflammation may offer protection against systemic infections, it can be detrimental in autoimmune diseases like SLE. In adaptive immunity, HSD suppresses Treg cells and activates Th17 cells, leading to increased inflammation and worsening autoimmune conditions [117]. Overall, the evidence suggests that high-sodium intake significantly contributes to the worsening of SLE in both murine and human models, primarily by enhancing systemic inflammation through various mechanisms. Table 4 [[110], [111], [112], [113],^115^,^116^]summarizes the impact of excessive sodium intake on lupus incidence and severity.

**TABLE 4:** The effect of excessive salt intake on lupus incidence and severity

Detailed study design	Organism	Interventions/nutrient	Outcome measured	Study result	Reference
Clinical trial	Human	Sodium intake	• 24-hour urinary sodium excretion • Frequency of T helper 17 (Th17) cells in peripheral blood • Function of Th17 cells in peripheral blood • Frequency of regulatory T cells (Treg) in peripheral blood • Function of regulatory T cells (Treg) in peripheral blood • Serum concentrations of cytokines (TGF-β and IL-9)	• A decrease in Th17 cells and an increase in Treg cells was observed. This trend reversed upon return to a normal-sodium diet. • The study also found reduced IL-9 concentrations in patients with SLE.	[113]
Cross-sectional	Human	—	• Anti-dsDNA concentration • Complement C4 concentration • Complement C3 concentration • hsCRP concentration	• Higher dietary sodium and lower dietary potassium intakes were associated with an increased risk of higher hsCRP in patients with SLE. • Dietary sodium intake was significantly associated with anti-dsDNA and complement C4 concentration. • Dietary potassium intake was associated with complement C3 concentration.	[115]
Observational	Human	—	• Food intake • Intestinal permeability using plasma zonulin • Gut microbiota • SLE Disease Activity (SLEDAI-2K) • C3 complement concentrations • C4 complement concentrations • C-reactive protein concentrations (CRP)	• Increased *Megamonas funiformis* abundance, elevated plasma zonulin, and higher sodium intake may contribute to reduced C3 complement concentrations in females with inactive SLE	[116]
*In vivo* trial	Mice	Sodium intake	• Survival rate • Disease severity • Frequencies of Th1 and Th17 cells • Ratios of Th1/Th2 and Th17/Treg • Serum TGF-β	• High-salt diet (HSD) worsens lupus nephritis (LN) in MRL/lpr mice, increasing disease severity and decreasing survival rate. • HSD increases the frequencies of Th1 and Th17 cells, and the ratios of Th1/Th2 and Th17/Treg. • High-salt treatment of CD4(+) T cells from patients with SLE and healthy donors increases Th17 cells, and this effect is reversed by SGK1 inhibitor.	[110]
*In vivo* trial	Mice	Sodium intake	• Plasma Anti-dsDNA Autoantibodies IhG • Urinary albumin • Mean arterial pressure • Albuminuria, urinary albumin excretion rate, urinary endothelin-1 (ET-1) excretion • Endothelin receptor protein expression (anti-ET-A, anti-ET-B) • Renal mRNA expression of NOS1, NOX2, MCP-1, TNF-α, SGK1, and IL-2	• Female NZBWF1 mice fed a HSD had increased circulating autoantibodies, but HSD did not significantly affect albuminuria or arterial pressure. • Urinary ET-1 excretion was increased, whereas renal endothelin A receptor and IL-2 expression were decreased in response to a HSD. • Chronic HSD may not accelerate cardiovascular and renal consequences commonly associated with SLE.	[112]
*In vivo* trial	Mice	Sodium intake	• Lupus progression • DC activation and cell ratios: MHC II, CD 80, CD 86 • DC Activation and maturation: CD11c, MHC II, CD 80, CD 86, CD 69, CD 40 • Division and proliferation indexes of T cells [antigen-presenting ability of DC(s)] • Production of autoantibodies: IgG, C3, anti-dsDNA concentrations, IgG1, IgG2a, IgG2b • Proinflammatory cytokines: IL-10, IL-17a, IFN-γ, IL-6, IL-4, TNF, IL-2 • Splenomegaly • Lymphadenopathy • Pathological renal lesions, interstitial fibrosis, and glomerular damage • Proteinuria concentrations • p38 MAPK–STAT1 pathway	• HSD accelerates the progression of murine lupus. • HSD increases the activation, maturation, and antigen-presenting ability of dendritic cells (DCs). • The p38 MAPK–STAT1 pathway plays an important role in NaCl-induced DC immune activities.	[111]

Abbreviations: DC, dendritic cell; ET-A, endothelin A receptor; ET-B, endothelin B receptor; LN, lupus nephritis; MAPK, mitogen-activated protein kinase; MCP-1, monocyte chemoattractant protein-1; MHC II, major histocompatibility complex class II; NOS1, nitric oxide synthase 1; NOX2, NADPH oxidase 2; SGK1, serum/glucocorticoid-regulated kinase 1; SLE, systemic lupus erythematosus; STAT1, signal transducer and activator of transcription 1; TGF-β, transforming growth factor beta; Th1, T helper 1; Th2, T helper 2; Th17, T helper 17; Treg, regulatory T cells.

### Vitamin D

Vitamin D, primarily recognized for its vital roles in calcium regulation, is also an important immune function regulator. Both the innate and adaptive arms of the immune system possess vitamin D receptors [118,119]. For example, upon pathogenic infection, innate immune cells, like macrophages, convert inactive vitamin D into its active form, calcitriol, which subsequently induces the production of antimicrobial peptides such as cathelicidin [120]. In addition, vitamin D also modulates the inflammatory cascade, such as by inhibiting proinflammatory cytokines like IL-6 and TNF-α, whereas upregulating anti-inflammatory mediators if overexpression of inflammation occurs [121]. In terms of adaptive immunity, vitamin D has been found to regulate T helper 1 (Th1) and Th17 cell responses, both implicated in autoimmunity and inflammation, and modulate the regulatory T-cell (Treg) functions to preserve immune tolerance [[122], [123], [124]]. B-cell differentiation and antibody production are also regulated by vitamin D, further exemplifying its roles in immune response [125]. Clinical evidence suggests that vitamin D deficiency is linked to increased susceptibility to infections such as COVID-19, influenza, and tuberculosis [[126], [127], [128], [129]]. Given the importance of vitamin D in immune function regulation, it is not surprising that vitamin D has a vital role in autoimmune disorders, including lupus.

Patients with lupus have been consistently shown to have vitamin D deficiency. Cutillas-Marco et al. [130] investigated a relatively small group of patients with cutaneous lupus erythematosus (CLE) and found that the patients with CLE had higher odds of having vitamin D deficiency. Similar observation was also reported by García-Carrasco et al. [131]. They found that 126 of 137 patients with SLE had either vitamin D insufficiency or deficiency, where insufficiency was defined as serum 25-hydroxyvitamin D <30 ng/mL and deficiency as <10 ng/mL [131]. Furthermore, a systematic review and meta-analysis carried out by Islam et al. [11] analyzed 34 case-control studies comprising 2265 patients with SLE and 1846 healthy controls, found that an inadequate concentration of vitamin D was prominent in patients with SLE when compared with healthy controls.

In addition, a small group of patients with CLE and vitamin D insufficiency (serum 25-hydroxyvitamin D concentrations <30 ng/mL) or deficiency (concentrations <10 ng/mL) were found to have improved disease severity when treated with an oral vitamin D supplementation schedule. They received 1400 IU of cholecalciferol and 1250 mg of calcium carbonate daily for 40 d. This was followed by 2 tablets daily, each containing a fixed combination of 1250 mg of calcium carbonate and 400 IU of cholecalciferol, for 1 y [130]. On the other hand, Hayashi et al. [132] reported that vitamin D appeared to have no beneficial effect on patients with SLE. However, there were some significant limitations in this particular study. There was no information with regards to the doses of vitamin D administered and length of treatment. Other confounding factors, such as other therapeutics, such as immunosuppressants, which definitely played a factor, also could not be excluded [132].

The therapeutic benefits of vitamin D relate to the modulation of immune function, particularly in the inflammation-related pathways. In the lupus-prone NZBWF1 mice, the correction of vitamin D concentration attenuated lupus pathology progression, delayed the onset of proteinuria, and reduced the concentrations of anti-dsDNA autoantibodies [133]. In addition, vitamin D promotes the adoption of a regulatory phenotype in lymphocytes and consequently increases the expression of IL-10, regulatory CD4^+^ T cells, and IL-10-expressing B cells [133]. Vitamin D also ameliorates the impairment of endothelium-dependent vasorelaxation and the shift toward the expression of IFN-stimulated genes in patients With lupus [134].

Piantoni et al. [135] carried out a 2-y prospective study of 34 patients diagnosed with SLE. During the first year, an intensive cholecalciferol regimen was given to 16 patients, involving a 300,000 IU initial dose and a monthly maintenance dose of 50,000 IU, amounting to 850,000 IU for the year. On the other hand, 18 patients received a standard dose of 25,000 IU of cholecalciferol every month, amounting to 300,000 IU annually. In the following year, the treatment plans were swapped between the groups. They observed an increase in the total count of CD4+CD45RA+CCR7− T cells, while noting a significant decrease in CD8+CD28− T cells. The analysis of PBMCs from 8 patients after undergoing the intensive regimen also showed a reduction in the IFN-γ/IL-4 ratio in CD8+ T cells over the 12-mo period. These results suggested that vitamin D supplementation modified the phenotype of T cells in patients with SLE [135]. Furthermore, Franco et al. [136] carried out a systematic review and meta-analysis and found that vitamin D supplementation appeared to be beneficial in patients with SLE by reducing anti-dsDNA positivity. Table 5 [130,132,133,135,[137], [138], [139], [140], [141], [142], [143]] summarizes the beneficial effects of vitamin D on lupus incidence and severity.

**TABLE 5:** Summarizes the beneficial effects of vitamin D on lupus incidence and severity

Detailed study design	Organism	Interventions/nutrient	Outcome measured	Study result	Reference
Cross-sectional and observational	Human	Oral vitamin D supplementation of cholecalciferol vitamin D-3 and calcium carbonate	• Serum 25-hydroxyvitamin D [25(OH)D] Concentrations • Cutaneous Lupus Erythematosus Disease Area and Severity Index (CLEDASI) • Number of exacerbations • Duration of active lesions • Patient assessment	• Vitamin D deficiency is more prevalent in patients with CLE than in healthy controls. • Increasing age and disease duration are associated with higher odds of having vitamin D deficiency. • Treating vitamin D insufficiency is associated with improved disease severity according to physician and patient assessments.	[130]
*In vitro*	Human	Oral vitamin D3 supplementation	• Externalization of neutrophil elastase (NE) during NETosis • Myeloperoxidase (MPO) absorbance during NETosis • Early apoptosis of endothelial cell • Late apoptosis of endothelial cell	• Vitamin D 1,25(OH)2D3 can reduce endothelial damage by decreasing NETosis activity in patients with SLE with hypovitamin D. • Significant decrease in early apoptosis was found with 10 nM of 1,25(OH)2D3 compared with control group. • Moderate positive correlation between NE externalizations with early apoptosis was found	[137]
Observational	Human	Vitamin D supplementation	• Body composition • Bone mineral density (BMD)	• The decrease in BMD observed in adolescents with juvenile SLE (JSLE) has been found to be correlated with the absence of vitamin D supplementation.	[138]
Randomized controlled trial	Human	Oral vitamin D3 supplementation	• Flow-mediated dilation (FMD) • 25(OH) vitamin D (25(OH)D) concentrations	• Half of patients with SLE with 25(OH)D concentrations <20 ng/mL who achieved 25(OH)D concentrations of ≥32 ng/mL experienced increases in FMD. • Those with increases in FMD had significantly higher final 25(OH)D concentrations. • Future studies designed to test the effect of repleting 25(OH)D on FMD in vitamin D-deficient patients with SLE will require 35 patients in each group.	[139]
Randomized controlled trial	Human	Oral vitamin D3 supplementation	• Interferon (IFN) signature	• Vitamin D supplementation significantly reduced the IFN signature in patients with SLE. • Vitamin D supplementation was well tolerated and had no adverse effects. • Vitamin D supplementation may be a potential therapeutic option for SLE.	[140]
Randomized controlled trial	Human	Oral vitamin D3 supplementation	• 25-OH vitamin D concentrations • Disease activity (SLEDAI) • SLE serology • Bone metabolism markers	• IR of vitamin D supplementation was found to be safe and effective in raising vitamin D serum concentrations in patients with SLE. • No significant differences in disease activity and SLE serology were found between the standard and intensive regimens. • No changes in mineral metabolism were observed.	[141]
Randomized controlled trial	Human	Oral vitamin D3 supplementation	• Visual Analog Scale (VAS) Scores of pain perception • Serum concentrations of leukotriene B4 (LTB4) • Serum concentrations of IL-6 • Serum concentrations of TNF-α • Serum concentrations of prostaglandin E2 (PGE2)	• Adding 4000 IU of vitamin D to analgesic regimens in patients with musculoskeletal pain led to a faster decline in VAS scores and a decrease in concentrations of inflammatory and pain-related cytokines. • The need for analgesic “rescue therapy” was significantly lower among the vitamin D-treated group. • TNFα and PGE2 concentrations decreased by 54.3% and 39.2%, respectively, in the group treated with vitamin D.	[142]
Prospective	Human	Oral vitamin D-3 supplementation	• Phenotypic analysis of peripheral T lymphocyte • Quantification of cytokine production from PBMCs • Number of Treg cells • Total amount of CD4+CD45RA+CCR7− T cells • Reduction of cd8+cd28− T cells • Reduction of the IFN-γ/IL-4 ratio among CD8+ T cells	• Vitamin D supplementation in patients with SLE resulted in an increase in the number of Treg cells and the total amount of CD4+CD45RA+CCR7− T cells, and a reduction of CD8+CD28− T cells. • Analysis of PBMCs from 8 patients following the intensive regimen showed a reduction of the IFN-γ/IL-4 ratio among CD8+ T cells after 12 mo. • Vitamin D supplementation may enhance Treg cells and the production of Th2 cytokines in patients with SLE.	[135]
Case report	Human	Medical nutrition therapy and oral vitamin D-3 supplementation	• Malnutrition (ASPEN criteria) • BMI, Fat Free Mass Index, Skeletal Mass Index (SMI), waist circumference • Patient’s functional capacity (Karnofsky Score) • LupusQoL • Nutritional intake: macronutrients, fat composition intake, SFAs, MUFAs	• Medical nutrition therapy (MNT) is an important part of comprehensive management of SLE. Nutritional supplementation includes “vitamin B complex 1 tablet t.i.d, folic acid 0.5 mcg q.d, calcium carbonate 500 mg t.i.d, and omega 3 fatty acid 1000 mg t.i.d. Cholecalciferol 6000 IU/day.” • Long-term corticosteroid use in patients with SLE can lead to malnutrition, sarcopenia, hypovitaminosis D, hypertension, and obesity. • Vitamin D supplementation combined with MNT improved nutritional status and quality of life in patients with SLE.	[143]
Cross-sectional analysis	Human	Oral vitamin D supplementation	• Systemic Lupus International Collaborating Clinics/American College of Rheumatology Damage Index (SDI) • Total SDI • SLE Disease Activity Index (SLEDAI)	• Patients with vitamin D supplementation were younger, received higher doses of prednisolone, and had higher estimated glomerular filtration rates than those without supplementation. • Disease-related SDI, total SDI, and SLEDAI did not significantly differ between patients receiving and not receiving vitamin D supplementation.	[132]
*In vivo* trial	Mice	Oral vitamin D supplementation	• Overall survival • Onset of proteinuria • Concentrations of antidouble-stranded DNA (anti-dsDNA) autoantibodies • IL-10-expressing CD4+ T cells • Regulatory CD4+ T cells • IL-10-expressing B cells	• Low dietary vitamin D intake accelerates lupus progression, reflected in reduced overall survival and an earlier onset of proteinuria, as well as higher concentrations of anti-dsDNA autoantibodies. • Low VD intake consistently hampered the adoption of a regulatory phenotype in lymphocytes, significantly reducing both IL-10-expressing and regulatory CD4+ T cells. • Low VD intake did not have consistent effects on the phenotype and function of innate immune cells.	[133]

Abbreviations: 25(OH)D, 25-hydroxyvitamin D; ASPEN, American Society for Parenteral and Enteral Nutrition; BMD, bone mineral density; CD, cluster of differentiation; CLE, cutaneous lupus erythematosus; CLEDASI, Cutaneous Lupus Erythematosus Disease Area and Severity Index; FMD, flow-mediated dilation; IFN, interferon; JSLE, juvenile systemic lupus erythematosus; LTB4, leukotriene B4; MNT, medical nutrition therapy; MPO, myeloperoxidase; NE, neutrophil elastase; NETosis, neutrophil extracellular trap formation; PBMCs, peripheral blood mononuclear cells; PGE2, prostaglandin E2; SDI, Systemic Lupus International Collaborating Clinics/American College of Rheumatology Damage Index; SLE, systemic lupus erythematosus; SMI, skeletal muscle index; Treg, regulatory T cells; VAS, visual analog scale; VD, vitamin D.

### Natural products

Conventional treatments for SLE, such as antimalarials, corticosteroids, and immunosuppressants, target the symptoms and underlying inflammation but can come with significant side effects [144]. Given the complexity of SLE and the potential adverse effects of standard therapies, there has been growing interest in the use of natural products as complementary or alternative treatments. These products, often derived from plants, herbs, animals, and other natural sources, have been used for centuries in traditional medicine systems worldwide. Table 6 [14,64,[145], [146], [147], [148], [149], [150], [151], [152], [153], [154], [155], [156], [157], [158], [159], [160]] summarizes the effects of natural products on lupus incidence and severity.

**TABLE 6:** The effects of natural products on lupus incidence and severity

Detailed study design	Organism	Interventions/nutrient	Outcome measured	Study result	Reference
Case control	Human	Polyphenols from oranges and apples	• Association between flavone intake and Blautia in SLE Group • Association between flavanones intake and Lactobacillus in SLE group • Association between dihydrochalcones intake and Bifidobacterium in SLE group • Association between dihydroflavanols intake and Faecalibacterium in control group • Association between flavanol intake and Bifidobacterium in control group • Association between orange intake	• Positive associations between flavone intake and Blautia, flavanones and Lactobacillus, and dihydrochalcones and Bifidobacterium were found in the SLE group. • Dihydroflavonols were directly associated with Faecalibacterium, whereas flavanol intake was inversely associated with Bifidobacterium in the control group. • Orange intake was directly associated with Lactobacillus and apple with Bifidobacterium in SLE, whereas red wine was the best contributor to Faecalibacterium variation.	[14]
Retrospective cohort	Human	Lycopene	• Mortality	• Higher serum lycopene has a protective effect on mortality in patients with SLE	[145]
Clinical trial	Human	Phenolic fraction (PE) of extra virgin olive oil (EVOO)	• Frequency of CD69+ Cells • Secretion of IFN • Secretion of TNF Α • Secretion of IL-6 • Secretion of IL-1Β • Secretion of IL-10 • Expression of I κB-Α • Extracellular signal-regulated kinase phosphorylation	• PE modulates cytokine production and attenuates induced T-cell activation, probably through NF-κB signaling pathway.	[146]
Cross-sectional	Human	—	• Caffeine intake • SLE-related disease phenotype • SLE disease activity Index 2000 (SLEDAI-2K) • Cytokine serum concentrations (IL-6, IL-10, IL-17, IL-27, IFN-γ, and IFN-α)	• Caffeine intake was negatively correlated with SLE disease activity, as measured by SLEDAI-2K. • Patients with a low intake of caffeine had a higher prevalence of lupus nephritis, neuropsychiatric involvement, hematological manifestations, hypocomplementemia, and anti-dsDNA positivity. • Patients with a high intake of caffeine had lower serum concentrations of IFN-γ, IFN-α, IL-17, and IL-6.	[147]
Systematic review of randomized controlled trials	Human	Curcumin/turmeric supplementation	• Inflammation • Oxidative Stress • Blood urea nitrogen (BUN) • Creatinine • GFR • Serum albumin • Proteinuria • Lipid profile (TG, VLDL, cholesterol) • Fasting blood sugar, HbA1c • Alanine and aspartate aminotransferase (ALT and ALP) (liver function)	• Curcumin/turmeric supplementation had favorable effects on renal diseases, particularly in terms of inflammation and oxidative stress. • Curcumin/turmeric supplementation had no considerable positive impact on clinical outcomes of kidney diseases, apart from proteinuria. • No serious adverse effects were reported following curcumin/turmeric supplementation.	[148]
*In vivo* trial	Mice	Polysaccharide of large yellow croaker swim bladder (PLYCSB)	• Concentrations of serum inflammatory cytokine concentrations of IL-6 • Concentrations of serum inflammatory cytokine concentrations of IL-12 • Concentrations of serum inflammatory cytokine concentrations of TNF-α • Concentrations of serum inflammatory cytokine concentrations of IFN-γ • Serum creatinine (sCR) concentrations • BUN serum concentrations • TC serum concentrations • TG serum concentrations • TP serum concentrations	• High concentration (50 mg/kg dose) of PLYCSB reduced the concentrations of serum inflammatory cytokines compared with a low concentration (25 mg/kg dose) and control mice. • PLYCSB significantly induced inflammation in kidney tissues of mice by downregulating NF-κB-p65, TGF-β1, Fas, FasL and upregulating IκB-α. • PLYCSB showed a potential curative effect on lupus nephritis as a drug or functional food.	[64]
*In vivo* trial	Mice	Extract of Gentiana macrophylla Pall. (GM)	• GM root extract significantly reduced cholesterol-aggravated apoptosis of the left ventricle in NZB/WF1 mice. • GM suppressed both intrinsic and extrinsic apoptotic pathways. • GM increased cardiac insulin-like growth factors (IGF)-1 survival signaling and anti-apoptotic proteins in LV tissues.	• GM root extract significantly reduced cholesterol-aggravated apoptosis of the left ventricle in NZB/WF1 mice. • GM suppressed both intrinsic and extrinsic apoptotic pathways. • GM increased cardiac IGF-1 survival signaling and antiapoptotic proteins in LV tissues.	[149]
*In vivo* trial	Mice	Isogarcinol	• Proteinuria • Serum biochemical indicators • Amount of serum antibodies • Renal histopathology score • Activation of CD4 T cells • Expression of inflammatory genes and cytokines in the kidneys and peritoneal macrophages	• Oral administration of isogarcinol (60 mg/kg) significantly reduced proteinuria, corrected abnormal serum biochemical indicators, and decreased the amount of serum antibodies and renal histopathology score. • Isogarcinol alleviated the abnormal activation of CD4 T cells and decreased the expression of inflammatory genes and cytokines in the kidneys and peritoneal macrophages. • The mechanism of action of isogarcinol is associated with downregulation of CD4 T cells and inflammatory effects.	[150]
*In vivo* trial	Mice	Extra virgin olive oil (EEVO) diet	• Kidney damage • Kidney expression of pro- and anti-inflammatory biomarkers, serum concentration • MMP-3 and splenocyte production of proinflammatory cytokines	• EEVO diet reduces kidney damage, kidney expression of proinflammatory biomarkers (PGE2), kidney activation of proinflammatory pathways (JAK/STAT, MAPK, and NF-κB), serum MMP-3 concentration, splenocyte expression of proinflammatory cytokines. • EEVO intake increases kidney expression of anti-inflammatory biomarkers (Nrf-2, HO-1)	[151]
*In vivo* trial	Mice	Lactoferrin	• Concentrations of antioxidant • Concentrations of inflammatory biomarkers • Fibrotic-related molecules in liver and serum.	• Lactoferrin increases antioxidant concentration in liver and serum, decreases proinflammatory indices and fibrotic-related molecules in liver.	[152]
*In vivo* trial	Mice	Diets supplemented with hydroxytyrosol (HTy) and hydroxytyrosyl acetate (HTy Ac)	• Cytokines concentrations • Renal changes of inflammatory markers • Signaling pathways	• Dietary phenol supplementation significantly reduced proinflammatory cytokines and prevented renal damage with a considerably blockage of different inflammatory-related pathways suggesting that HTy and HTy Ac supplementation might provide a basis for developing a new dietary strategy for prevention and management of SLE.	[153]
*In vivo* trial	Mice	Curcumin	• Arthritis score • Proteinuria concentration • Body weights • Adaptive immune system components (Th1, Th2, Th17, and Treg percentages) • Proinflammatory (cytokines IL-6 and IFN-Α) • ANA	• Decreased arthritis score, proteinuria concentrations, Th1, Th2, and Th17 percentages, as well as serum IL-6, IFN-α, and ANA concentrations, whereas Treg percentages showed a slight increase	[154]
*In vivo* trial	Mice	Diets enriched with oleuropein and its new derivate, peracetylated oleuropein	• Renal histology • MMP-3 serum concentrations • iNOS/β-Actin, mPGEs-1/β-Actin, PGE2 protein expression in kidneys • Upregulation and densitometry analysis of Nrf2, HO-1, β-actin, pSTAT3, NFkB-p65, IkB-α, NLRP3, ASC, IL-18, procaspase-1, cleaved caspase-1, procaspase-11, part-cleaved caspace-11, cleaved caspase-11	• Dietary oleuropein (OL) and its new derivate, peracetylated oleuropein (Per-OL), attenuated murine lupus nephritis. • OL and Per-OL increased the expression of antioxidant proteins HO-1 and Nrf2. • OL and Per-OL suppressed the activation of JAK/STAT, MAPK, NF-κB, and NLRP3 inflammasome pathways.	[155]
*In vivo* trial	Mice	Oral curcumin administration	• Body weight and composition (body fat %) • Spleen weight • Circulating dsDNA autoantibodies • B lymphocytes (CD45R+ Cells) • Renal injury (albumin excretion, glomerulosclerosis score, BUN) • Hemodynamic function GFR, mean arterial pressure (MAP)]	• Oral administration of curcumin attenuates autoimmunity and renal injury in female NZBWF1 mice with SLE. • Curcumin treatment reduced spleen weight and glomerulosclerosis when treatment started at 26 wk of age. • When curcumin treatment started at 32 wk of age, renal injury (glomerulosclerosis, BUN) was reduced in SLE mice compared with vehicle-treated SLE mice.	[156]
*In vivo* trial	Mice	Curcumin	• Proteinuria • BUN • Serum creatinine • Glomerulonephritis (GN Score) • Crescent formation • Tubule-interstitial pathology • Lymphocytic infiltration • Renal lymphoid cell infiltration: CD3+ cells, B220+ cells, CD11b+ cells, CD11c+ cells • Phosphorylation cell signaling: NF-κB, P38, Erk1,2 and Bad, AKT, IκB and Bcl-2 Serum autoantibody concentrations • Splenomegaly (spleen weight, splenocyte, splenic B and T cells, macrophage, dendritic cells, populations, and activation status) • Autoantibody production: IgG anti-dsDNA, antihistone, and anti-ssDNA, and IgM anti-dsDNA, anti-ssDNA, and antihistone • Complete blood count: WBC, RBC, HGB, HCT, MCV, MCHC, RDW, alanine aminotransferase activity, aspartate aminotransferase activity	• Curcumin treatment reduced proteinuria, blood urea nitrogen, glomerulonephritis, crescent formation, tubule-interstitial disease, and renal infiltration by lymphocytes in both the anti-GBM and MRL.lpr mouse models. • Curcumin treatment reduced activation of the NFkB, MAPK, AKT and pBAD pathways either systemically, or within the inflamed kidneys. • Curcumin ameliorated kidney disease in the 2 mouse models with either acute or chronic nephritis.	[157]
*In vivo* trial	Mice	Orally administered amaranth oil	• IgG and IgM histone autoantibody absorbance concentrations • IgG dsDNA, ssDNA, and nucleosome autoantibody absorbances • IgM dsDNA, ssDNA, and nucleosome autoantibody absorbances • Splenic immune cell populations	• Mice receiving amaranth oil showed decreased IgG and IgM histone autoantibody absorbance concentrations throughout the study. • IgG dsDNA, ssDNA, and nucleosome autoantibody absorbances were lower than those of the control group for the first 42 d. • IgM dsDNA, ssDNA, and nucleosome autoantibody absorbances were lower only for the first 14 d. • “There were no significant differences found among the splenic immune cell populations tested between the control and experimental groups.”	[158]
*In vivo* trial	Mice	Oleocanthal (OLE) supplemented diet	• Renal damage • Aortic endothelial dysfunction • Cytokine concentrations (IL-17, TNF-α, IL-1β, IL-6, and IFN-γ) • Presence of immunoglobulin (Ig) G and IgM immune complexes • Signaling pathways and oxidative-inflammatory-related mediators	• Dietary OLE supplementation reduced Th1/Th17 proinflammatory cytokines production and alleviated renal damage by decreasing immunoglobulin complexes deposition, and inflammation-mediating enzymes expression. • Dietary OLE improved aortic endothelial dysfunction and vascular reactivity, normalizing endothelial nitric oxide synthase (eNOS) uncoupling, and NADPH oxidase-1 (NOX-1) overexpression.	[159]
*In vitro* trial	Human and mice	dietary taurine	• Serum amino acid concentrations of metabolism in patients with SLE: Taurine, L-Histidine, L-Phenylalanine, L-Tryptophan • Expression of: IFN-α, IFN-β, TNF-α, ifit1 mRNA, ifr7 mRNA, mx1 mRNA, oas 1 mRNA, anti-dsDNA • Proteinuria concentrations • IgG concentrations • ROS concentrations • NADP+/NADPH concentrations • MHC II and CD 86 expression concentrations • B cell and T-cell count and Activity • SLEDAI	• Metabolic abnormalities in SLE can dysregulate multiple immune cells. • Supplement of taurine promoted IFN-I induced genes’ expression, activated lymphocyte, increased autoantibodies, and proteinuria, leading to more serious nephritis. • Taurine metabolism can aggravate the progression of lupus by promoting the function of plasmacytoid dendritic cells.	[160]

Abbreviations: AKT, protein kinase B; ALT, alanine aminotransferase; ANA, antinuclear antibody; ASC, apoptosis-associated speck-like protein containing a caspase recruitment domain; CD, cluster of differentiation; dsDNA, double-stranded DNA; eNOS, endothelial nitric oxide synthase; EVOO, extra virgin olive oil; FasL, Fas ligand; GFR, glomerular filtration rate; GM, Gentiana macrophylla; HbA1c, glycated hemoglobin; HCT, hematocrit; HGB, hemoglobin; HO-1, heme oxygenase-1; HTy, hydroxytyrosol; HTy Ac, hydroxytyrosyl acetate; IFN, interferon; IGF-1, insulin-like growth factor 1; JAK/STAT, Janus kinase/signal transducer and activator of transcription; LN, lupus nephritis; MAPK, mitogen-activated protein kinase; MCHC, mean corpuscular hemoglobin concentration; MCV, mean corpuscular volume; MHC, major histocompatibility complex; MMP-3, matrix metalloproteinase-3; NF-κB, nuclear factor kappa B; NLRP3, NOD-like receptor family pyrin domain containing 3; NOX-1, NADPH oxidase-1; OLE, oleocanthal; PBMCs, peripheral blood mononuclear cells; PE, phenolic fraction; PGE2, prostaglandin E2; PLYCSB, polysaccharide of large yellow croaker swim bladder; RBC, red blood cell; RDW, red cell distribution width; ROS, reactive oxygen species; SLE, systemic lupus erythematosus; SLEDAI, Systemic Lupus Erythematosus Disease Activity Index; SLEDAI-2K, Systemic Lupus Erythematosus Disease Activity Index 2000; ssDNA, single-stranded DNA; STAT, signal transducer and activator of transcription; TC, total cholesterol; TGF-β, transforming growth factor beta; TG, triglycerides; Th, T helper; WBC, white blood cell.

### Olive oil

Virgin olive oil (VOO) and extra virgin olive oil (EVOO) are both essential components of the Mediterranean diet. Olive oils are well recognized for their anti-inflammatory and immunomodulatory properties, which are attributable to their diverse range of bioactive [161]. The health benefits of olive oils are traditionally ascribed to their major component (MUFAs, mainly oleic acid) [162]. However, more recent evidence indicates that minor components of olive oils, which include polyphenol fractions (constituting up to 2% of total content), also contribute to their health-beneficial properties [163]. This growing understanding fuels the interest in integrating olive oil into daily diets as an adjunct therapy in managing autoimmune diseases.

Cells of the innate immune system, including macrophages and monocytes, play a crucial role in initiating and guiding the adaptive immune response during inflammation. In patients with SLE, monocytes and macrophages display altered phenotypes, characterized by an overproduction of proinflammatory cytokines. Aparicio-Soto et al. [151] conducted a study to explore the potential benefits of a diet containing VOO in modulating immune-inflammatory responses in SLE. They randomly assigned 60 female BALB/c mice, a commonly used inbred albino strain, into 4 experimental groups: mice injected with pristane (to induce a lupus-like disease) and fed a diet of either VOO or sunflower oil, and mice injected with a saline solution and given a diet of either VOO or sunflower oil. Notably, the release of nitrite and proinflammatory cytokines (IL-6, IL-17, and TNF-α) by LPS-activated peritoneal macrophages from the pristane-SLE mice was significantly lower in those fed with the VOO diet compared with those on the sunflower oil diet [151]. Furthermore, the same group extracted the phenolic fraction (PF) from VOO and studied its effect on LPS-treated human monocytes [164]. Treatment with LPS downregulated the expression of the anti-inflammatory PPARγ, and upregulated the expression of the proinflammatory IL-6, IL-17, TNF-α, as well as toll-like receptor 4 in monocytes. However, this effect was counteracted by PF from VOO in a dose-dependent manner. PF from VOO also blocked the genetic signature of the proinflammatory M1 macrophages while favoring the phenotype of anti-inflammatory M2 macrophages upon canonical polarization of naïve monocyte-derived macrophages [164].

The adaptive immune cells also play a major role in the pathogenesis of SLE. One of the immunological characteristics of patients with SLE is an altered T-cell response manifested by an imbalance of the production of cytokines like IL-6, IL-1, IL-10, and TNF-α [165]. In addition, T-cell activation, characterized by increased CD69 expression, is commonly increased in patients with SLE [47]. To elucidate the effect of PF from EVOO on T-cell activation and cytokine release, Aparicio-Soto et al. [153] isolated the PBMCs of patients with SLE and healthy controls. Notably, their findings revealed that, although stimulation with phytohemagglutinin significantly increased the activation status of peripheral blood CD4^+^ T cells (as evidenced by the expression of the CD69 surface marker), the introduction of PF from EVOO (5 and 10 μg/mL) reduced the frequency of CD69^+^ cells among CD4^+^ T cells in both groups after 24 h of cell culture, in a dose-dependent manner [146]. Additionally, PF from EVOO also lowered the production of phytohemagglutinin-induced proinflammatory cytokines such as IL-6, IL-1β, IFN- γ, and TNF-α in the PBMCs of both healthy individuals and patients with SLE.

Numerous animal studies have investigated the potential effects of a diet rich in EVOO and diets that include specific phenolic compounds such as oleuropein, oleocanthal, and hydroxytyrosol on mitigating the inflammatory and oxidative damage associated with lupus nephritis. For instance, the generation of reactive oxygen species and a compromised antioxidant response have been identified as contributors to renal damage in lupus [166]. In light of this, diets containing EVOO [151], oleuropein [155], oleocanthal [159], and hydroxytyrosol [153] have demonstrated their ability to upregulate the expression of antioxidant proteins, namely Nrf-2 and HO-1, while simultaneously decreasing the production of the proinflammatory proteinE2 (PGE2), in the kidneys of the pristane-induced SLE mouse model. Histological examinations further indicate that supplementing with EVOO and specific phenolic compounds can reverse various kidney abnormalities, such as interstitial fibrosis, thyroidization, and abundant presence of inflammatory mononuclear cells in the renal interstitium, which are seen in the pristane-induced SLE mouse model, albeit to varying extents [151,155,159].

### Curcumin

Curcumin is the active ingredient in turmeric, a commonly used spice in Asian cuisines, especially Indian dishes. It is a polyphenol with antioxidant, anti-inflammatory, and potential anticancer properties [167]. Research spanning both human and animal models has highlighted its therapeutic potential for autoimmune diseases, including multiple sclerosis, RA, and inflammatory bowel disease [168].

Of particular interest is its role in SLE. In pristane-induced SLE mice model, a 16-wk daily curcumin regimen at doses of 12.5, 50, and 200 mg/kg led to a simultaneous dose-dependent decrease in Th1 and Th2 [154]. Concurrently, the study revealed a decline in the Th17 cell population and Th17/Treg ratios. Additionally, curcumin suppressed the production of proinflammatory cytokines, IL-6 and TNF-α, both implicated in the progression of SLE [168] and reduced arthritis score and lowered ANA [154].

Lupus nephritis is one of the most severe lupus manifestations. Studies using murine models have provided valuable insights into curcumin’s potential in alleviating lupus nephritis. A study on the NZBWF1 model demonstrated a decrease in spleen weight, plasma blood urea nitrogen (BUN), and glomerulosclerosis score upon a 2-wk administration of curcumin at 500 mg/kg [156]. However, there was no significant effect on albuminuria, circulating CD45R^+^ B cells, IgG anti-dsDNA, and glomerular filtration rate (GFR). The study’s short duration might have contributed to these outcomes. On the other hand, a longer 2-mo regimen with curcumin at 1000 mg/kg in the MRL.*lpr* model not only significantly reduces BUN, spleen weight, and glomerulonephritis score, but also proteinuria along with IgG anti-dsDNA, IgG anti-ssDNA, IgG antihistone, and IgM antihistone [157]. This suggests that curcumin possesses therapeutic potential for SLE-related kidney pathologies across varying durations of treatment. The brief 2-wk treatment highlights curcumin’s immediate therapeutic capabilities, whereas the extended 2-mo study underscores its preventative potential.

Although animal studies provide insights into curcumin’s effects on clinical parameters of lupus nephritis, their translation to human contexts reveals some inconsistent results. Indeed, a systematic review of randomized controlled trials involving 631 patients with renal disorders, including lupus nephritis, highlighted that turmeric/curcumin supplementation showed positive effects on inflammatory and oxidative stress markers [148]. However, apart from proteinuria, their influence on key clinical markers like BUN, creatinine, GFR, and serum albumin was limited [148]. These differences highlight the importance of additional research in human clinical settings.

### Other natural extracts

Beyond EVOO and curcumin, several other less-researched natural extracts also exhibit potential benefits in treating SLE. In NZBWF1 lupus mice model fed with a high-cholesterol diet, lactoferrin (a glycoprotein found in colostrum and milk) demonstrated ameliorative effects on hepatic fibrosis by inhibiting TGF-b/Smad fibrotic signaling [152]. Similarly, the root extract of *Gentiana macrophylla Pall.* (commonly known as qin jiao) alleviates cholesterol-aggravated cardiac apoptosis, potentially by augmenting the cardiac IGF-1 survival signal through the phosphorylation of PI3K and AKT and the inhibition of both extrinsic and intrinsic apoptosis signals [149]. Other natural extracts that have shown therapeutic benefits in murine lupus models include amaranth oil [115,158], isogarcinol [150], and polysaccharide of large yellow croaker swim bladder [64].

In a retrospective cohort study, higher serum lycopene (a carotenoid hydrocarbon found in tomatoes and other red fruits and vegetables) of participants with SLE was associated with lower mortality [145]. On the other hand, a cross-sectional study of patients with SLE identified associations between caffeine intake and lower disease activity and cytokine concentrations [147]. However, not all-natural extracts manifest positive outcomes. For example, metabolomic profiling showed elevated taurine concentrations in serum of patients with SLE [160]. Moreover, taurine supplementation in mice was observed to exacerbate the progression of the lupus condition [160].

### General discussion

**FIGURE 2:**
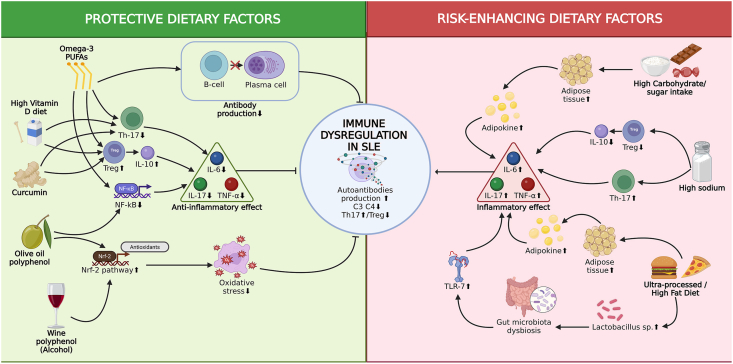
The summary of the effects of different dietary and nutritional factors in influencing the risks of lupus incidence and severity.

The primary focus of this review was to analyze the relationships between dietary and nutritional factors and lupus pathophysiology (Figure 2). Several factors may lower lupus risk, including diet quality, vitamin D, ω-3 fatty acids, alcohol consumption, and natural products. A strong link exists between high-quality diets and lower lupus incidence and severity. Poor dietary patterns, such as Western or high-salt diets, promote excess weight and inflammation. Several studies show an association between higher BMI and lupus disease activity (Table 1). Essential fatty acids ω-3 and ω-6 are key in maintaining immune homeostasis. ω-3 fatty acids produce anti-inflammatory mediators, whereas ω-6 fatty acids produce proinflammatory mediators. They regulate inflammatory balance, antigen presentation, T-cell and B-cell activity, and leukocyte recruitment in lupus. Higher ω-3 and lower ω-6 concentrations support a more favorable immune state (Table 2).

Alcohol intake has been associated with lower lupus incidence. However, it remains unclear whether this is due to alcohol itself or other compounds such as phenolics in wine. Still, alcohol may be beneficial by lowering circulating SCF (Table 3). In contrast, high-sodium intake appears to worsen lupus severity by promoting a proinflammatory state, including increased Th1:Th2 and Th17:Treg ratios and higher anti-dsDNA IgG and C4 concentrations (Table 4).

Among micronutrients, vitamin D is particularly important for reducing symptoms and modulating disease progression. Adequate vitamin D concentrations can delay proteinuria and reduce anti-dsDNA autoantibodies, key markers of disease activity. It also regulates IL-10, CD4+ Treg cells, and T-cell phenotypes, promoting a more balanced immune response. These findings suggest vitamin D may serve as both a prognostic biomarker and a therapeutic agent (Table 5). Natural compounds such as olive oil (rich in phenolics) and curcumin may also provide benefits due to their antioxidant and immunomodulatory effects (Table 6).

## Strengths and Limitations of Evidence

A principal strength of this scoping review is its deliberate and systematic inclusion of evidence spanning the entire translational research continuum from *in vitro* mechanistic studies through animal models to human observational studies and clinical trials. This approach, while inherently heterogeneous, offers several distinct advantages. First, mechanistic exploration**.** By incorporating *in vitro* and animal studies, we discuss insights into the biological plausibility and potential mechanisms underlying observed associations in human studies. For example, while human studies demonstrate that ω-3 fatty acids are associated with reduced disease activity [82,82], animal studies elucidate the underlying immunological mechanisms, including suppression of CD4+ T-cell-related genes [90], inhibition of B-cell and T-cell proliferation [86], and downregulation of IFN-regulated genes [87]. This mechanistic grounding strengthens confidence in the causal nature of these associations.

Second, hypothesis generation**.** The inclusion of preclinical studies identifies promising dietary factors such as olive oil phenolics [151,164], curcumin [154,157], lactoferrin [152], and *Gentiana macrophylla* extract [149] that have limited human evidence but warrant future investigation.

Third and arguably most importantly: identification of knowledge gaps. Mapping the full evidence landscape reveals critical discontinuities between preclinical promise and clinical validation. For instance, although curcumin shows robust effects in multiple murine lupus models, human evidence remains limited to renal outcomes with inconsistent findings. This gap identification is a core objective of scoping reviews and provides clear direction for future research. The strongest evidence for clinical application comes from human RCTs and large prospective cohort studies. Evidence from animal models should be interpreted as mechanistic and exploratory, providing biological plausibility and generating hypotheses for future human investigation rather than supporting immediate clinical recommendations.

Several factors constrain the translation of animal and *in vitro* findings into human clinical practice. Lupus in murine models is typically induced experimentally (e.g., pristane, crystalline silica), or genetically driven (e.g., NZB/W F1, MRL/lpr). These models are inherently biased toward specific immunopathological pathways and therefore do not fully recapitulate the clinical, genetic, and mechanistic heterogeneity of human SLE [169]. In addition, species-specific differences in immune regulation, metabolism, and microbiome composition may influence responses to dietary interventions [170,171]. Furthermore, translation of dosing from animal models to humans remains inherently challenging [172]. As such, it is often unclear whether doses used in human studies achieve biologically equivalent exposures relative to preclinical models. Taken together, although preclinical studies provide essential mechanistic insights and support causal inference, their translational value lies primarily in hypothesis generation rather than direct clinical extrapolation.

The strongest evidence for clinical application remains derived from human randomized controlled trials and large prospective cohort studies. Future research should prioritize well-designed human interventional studies to validate mechanistic findings and establish clinically relevant exposure levels. Furthermore, advances in humanized mouse models, in which immunodeficient mice are engrafted with components of the human immune system, may serve as complementary tools to refine hypotheses before clinical testing [173].

Despite these strengths, several limitations should be considered. Dietary components are unlikely to act in isolation; however, this review primarily summarizes evidence on individual nutrients, as the available literature on their combined effects remains limited. Some experimental studies in other inflammatory conditions (e.g., osteoarthritis) report greater protective effects with combined interventions of curcumin and vitamin D compared with individual components [174]. Evidence from a factorial randomized trial further showed that combined supplementation with vitamin D and ω-3 was associated with reduced autoimmune disease risk [175]. However, no significant interaction between the 2 interventions was observed, suggesting largely independent or additive effects rather than true synergy (i.e., where the effect of one intervention depends on the presence of the other) [175]. Overall, while combined dietary strategies may offer potential benefits, current evidence does not allow definitive conclusions regarding their nature or magnitude in SLE across most dietary factors. Future studies designed to evaluate multinutrient interventions are needed to better characterize their combined impact on disease risk and progression.

As a scoping review, we did not perform formal quality or risk-of-bias assessments for individual studies, limiting evaluation of the strength and reliability of the evidence. The lack of quantitative synthesis means pooled effect estimates cannot be provided. Although comprehensive, the thematic approach may oversimplify complex interactions between dietary components. The 2012 to 2023 search window may exclude older relevant studies, but it likely captures the most current and methodologically robust evidence, given rapid advances in nutrition and immunology.

The included studies show substantial heterogeneity across multiple dimensions, complicating synthesis and limiting precision. Restricting to English-language publications may introduce language bias, and publication bias is possible, as negative findings may be underreported.

Findings are sometimes contradictory, with studies reporting both positive and negative effects of individual nutrients. This may reflect the difficulty of isolating dietary effects from other lifestyle factors, or suggest that individual nutrients may be more influential than overall dietary patterns. It may also result from residual confounding in observational studies. Null findings from several high-quality studies indicate that associations between whole diets and SLE risk require further investigation. Therefore, more large-scale, diverse population studies and randomized controlled trials are needed to confirm these associations and inform dietary recommendations.

In conclusion, the present review illustrates a link between dietary and nutritional factors and lupus incidence and severity. The integration of nutritional and dietary guidance into the management of lupus represents a promising adjunct to conventional pharmacological treatments. The evidence collected in this review underscores the potential of specific dietary interventions to modulate immune function, reduce inflammation, and possibly decrease the frequency and severity of lupus flares. Future research should aim to establish more precise dietary recommendations and explore the mechanisms by which diet influences lupus activity. Large-scale, randomized controlled trials are needed to confirm the therapeutic potential of dietary and nutritional interventions and to integrate them effectively into the standard of care for lupus management.

## Author contributions

The authors’ responsibilities were as follows – JNJ, MP, NP: contributed to writing original draft, and review and editing; ID: contributed to writing original draft; FN: contributed to visualization; and FZ: contributed to conceptualization, methodology, writing original draft, review and editing, supervision, visualization, and funding acquisition.

## Data availability

Data described in the manuscript will be made available upon reasonable request.

## Funding

The article processing charge was funded by the Efi Propolis Research Fund.

## Declaration of Generative AI and AI-Assisted Technologies in the Writing Process

The authors declare that no generative AI or AI-assisted technologies were used in the writing of this manuscript.

## Conflict of interest

FZ is associated with Efi Propolis. All other authors declare no competing financial interests and no conflict of interest.
